# Interventions for anxiety in mainstream school‐aged children with autism spectrum disorder: A systematic review

**DOI:** 10.1002/cl2.1086

**Published:** 2020-05-05

**Authors:** Kylie Hillman, Katherine Dix, Kashfee Ahmed, Petra Lietz, Jenny Trevitt, Elizabeth O'Grady, Mirko Uljarević, Giacomo Vivanti, Darren Hedley

**Affiliations:** ^1^ Australian Council for Educational Research Camberwell Victoria Australia; ^2^ Australian Council for Educational Research Adelaide South Australia Australia; ^3^ Division of Child and Adolescent Psychiatry, Stanford Autism Center, Department of Psychiatry and Behavioral Sciences, School of Medicine Stanford University Palo Alto California; ^4^ A. J. Drexel Autism Institute Dornsife School of Public Health, Drexel University Philadelphia Pennsylvania; ^5^ Olga Tennison Autism Research Centre, School of Psychology and Public Health, College of Science, Health and Engineering LaTrobe University Bundoora Victoria Australia

## 1 PLAIN LANGUAGE SUMMARY

### 1.1 Cognitive behavioral therapy for anxiety in school‐aged children with autism spectrum disorder can reduce anxiety

Cognitive behavioral therapy (CBT) interventions to reduce the level of anxiety in students with autism spectrum disorder (ASD) are moderately effective.

### 1.2 What is this review about?

Anxiety is a common problem in school‐aged children with ASD. CBT and other psychosocial interventions have been developed as alternatives to pharmacological intervention to treat anxiety in students with ASD.

**What is the aim of this review?**

This Campbell systematic review examines the effects of interventions for reducing anxiety in school‐aged children with ASD, compared to treatment‐as‐usual. The review summarizes evidence from 24 studies using an experimental or quasi‐experimental design.

### 1.3 What studies are included?

Twenty‐four studies, involving 931 school‐aged children with ASD (without co‐occurring intellectual disability) and clinical anxiety, are summarized in this review. The studies were experimental or quasi‐experimental control‐treatment trials, deemed to be of sufficient methodological quality and with reduced risk of bias. Studies spanned the period 2005 to 2018 and were mostly carried out in Australia, the UK, and the USA.

Examined interventions ranged across clinical, school‐based, or home‐based settings, with group or individual treatment formats. Twenty‐two of the studies used a CBT intervention. One study used peer‐mediated theater therapy and one study examined the benefits of Thai traditional massage for reducing anxiety. Most interventions involved parents/caregivers and were conducted face‐to‐face.

### 1.4 What are the main findings of this review?

Overall, the effects of interventions on anxiety show a statistically significant moderate to high effect, compared to waitlist and treatment‐as‐usual control conditions at posttreatment.

However, effects differ depending on who reports on the student's anxiety. Clinician reports indicate a very high statistically significant effect, parent reports indicate a high significant effect, and self‐reports indicate only a moderate significant effect on the reduction of anxiety in students with ASD.

There are larger effects for treatments that involve parents than for student‐only interventions. Effects are also larger for individual one‐on‐one interventions compared to treatments delivered in a group with peers.

There are several risk‐of‐bias issues in most studies included in the review, mainly due to the unavoidable limitation that participants cannot be blinded to the treatment group, which may upwardly bias the estimated effects.

There are also limitations in the description of randomization in a third of the studies, so findings should be treated with caution.

### 1.5 What do the findings of this review mean?

The findings provide evidence in support of interventions, particularly CBTs, designed to reduce anxiety symptoms in school‐aged children with ASD.

These findings accord with and build upon the findings of previous systematic reviews into the effectiveness of interventions to reduce anxiety in children and youth with ASD. However, because of the risk of bias in current findings, it would be useful to have further studies with larger sample sizes and to reduce potential biases where possible.

### 1.6 How up‐to‐date is this review?

The review authors searched for studies up to the end of 2018.

## 2 EXECUTIVE SUMMARY/ABSTRACT

### 2.1 Background

Anxiety is a common problem in school‐aged children with ASD. CBT and other psychosocial interventions have been developed as alternatives to pharmacological intervention to treat anxiety symptoms in students with ASD without co‐occurring intellectual disability. This present synthesis of evidence is a systematic review and meta‐analysis examining the efficacy of interventions for reducing anxiety among school‐aged children with ASD.

### 2.2 Objectives

This review aims to address the question of what the relative effectiveness of interventions is for managing anxiety of school‐aged children with ASD in school, family, and clinical settings.

### 2.3 Search methods

The following databases were searched for references from 1996 up to 31 December 2018: EBSCO (including Academic Search Complete, British Education Index, CINHAHL, Education Research Complete, ERIC, PsychINFO, and SocINDEX), Informit (A + Education), Elsevier (including EMBASE and SCOPUS), PubMed and Proquest (CBCA Complete). We also searched the reference lists of published and unpublished literature papers, as well as gray literature sources, selected websites, trial registries, and experts in the field of autism to inquire about studies.

### 2.4 Selection criteria

Studies were included in the review if they met the following criteria.

1. The patient/client population was school‐aged children (5 to 18 years old) diagnosed with ASD (inclusive of autism, ASD, Autistic Disorder, Asperger's Disorder, Asperger Syndrome, atypical autism, and PDD‐NOS) by a professional eligible to diagnose these conditions, *and also* experiencing anxiety symptoms or a diagnosis of an anxiety disorder provided by a professional eligible to diagnose such conditions.
2. The intervention was focused on reducing anxiety symptoms and included at least one of the following seven elements: (a) psychoeducation, (b) exposure, (c) cognitive restructuring, (d) parent training or parent psychoeducation, (e) relaxation, (f) modeling, and (g) self‐monitoring.
3. At least one outcome measure was a standardized continuous measure of anxiety (parent, clinician or self‐reported).
4. The study was published between the years 1996 and 2018.

### 2.5 Data collection and analysis

Four authors independently selected and appraised studies for inclusion, while two authors evaluated the risk of bias in each subsequently included study. All outcome data were continuous, from which standardized mean difference effect sizes were calculated. We conducted random effects meta‐analysis, which means we assumed individual studies would provide different estimates of treatment effects. Where outlier studies were identified, analyses were repeated after the outlier had been removed from the list of studies. Analyses were conducted separately according to the respondent on the outcome measure of anxiety: clinician, parent or subject (child or youth). Moderator analyses were undertaken to examine differences in effect sizes depending on whether or not the family was involved and whether treatment occurred in groups or individually.

### 2.6 Results

Eighteen randomized controlled trials (RCTs) and six quasi‐experimental studies met the inclusion criteria. These studies evaluated the effects of interventions targeting anxiety in 931 (764 male and 167 females) participants aged 3–19 years. Overall, the effects of interventions on anxiety were statistically significant and of moderate to high effectiveness, compared to waitlist and treatment‐as‐usual control conditions at posttreatment (standardized mean difference after removal of outliers *SMD* = −0.71, 95% confidence interval [CI]: −0.97, −0.46; *z* = −5.42, *p* < .01), where SMDs of 0.05, 0.19, 0.45, and 0.70 were taken to be indicative of low, moderate, high, and very high effects, respectively. Results also suggested the reported effectiveness of treatment varied as a function of the informant on outcome measures—clinician reports indicate a very high statistically significant effect (*SMD* = −0.84, 95% CI: −1.15, −0.54; *z* = −5.43, *p* < .01), while parent reports indicate a high significant effect (*SMD* = −0.53, 95% CI: −0.76, −0.31; *z* = −4.73, *p* < .01). Results based on the subjects’ self‐reports indicated a moderate significant effect on the reduction of anxiety (*SMD* = −0.35, 95% CI: −0.55, −0.15; *z* = −3.41, *p* = .001).

Moderators indicated larger effects for treatments that involved parents (*SMD* = −0.74, 95% CI −1.06, −0.42; *z* = −4.55, *p* < .01) than for student‐only interventions (*SMD* = −0.60, 95% CI −1.03, −0.17; *z* = −2.73, *p* < .01). Treatments that were administered individually one‐on‐one (*SMD* = −1.24, 95% CI −1.75, −0.74; z = −4.87, *p* < .01), indicated larger effects than for treatments delivered in a group context with peers (*SMD* = −0.37, 95% CI −0.54, −0.19; *z* = −4.10, *p* < .01).

No adverse events were reported. Given the nature of the interventions and the selected outcome measures, the risk of performance and detection bias are generally high, particularly for those studies that used outcome measures based on parent and self‐reports.

### 2.7 Authors’ conclusions

There is evidence that CBT is an effective behavioral treatment for anxiety in some children and youth with ASD without co‐occurring intellectual disability. Evidence for other psychoeducational interventions is more limited, not just due to the popularity of CBT but also due to the quality of the smaller number of non‐CBT studies available.

While there is evidence that CBT is an effective behavioral treatment for anxiety in some children and youth with ASD, work remains to be done in terms of identifying the characteristics of these interventions that contribute to their effectiveness and identifying the characteristics of participants who are more likely to respond to such interventions.

## 3 BACKGROUND

### 3.1 The condition

ASD refers to a group of neurodevelopmental disorders characterized by difficulty with communication and social interaction, and the presence of restricted, rigid, and routinized patterns of behaviors and interests (American Psychiatric Association, 2013). These symptoms appear on a continuum (or spectrum), with some children experiencing relatively mild symptoms, while others experience quite severe symptomatology. Notwithstanding the changing ways in diagnosing children with ASD, the reported prevalence appears to be increasing over time (ABS, 2016; Fombonne, 2018). For example, the current rate of prevalence in the United States (US) has reportedly risen by 15% over recent years to 1 in 59 (Autism Speaks, 2016; Baio et al., 2018; CDC, 2012), while in Australia, the rate has increased by 42% between 2012 and 2016, to 1 in 150 children (ABS, 2016).

In addition to increasing numbers, recent research shows that the number of students with ASD attending mainstream schools is also increasing (Zainal & Magiati, 2016). The exact reason for the increase in prevalence is unclear, but may be related to changes in the Diagnostic and Statistical Manual of Mental Disorders (DSM; American Psychiatric Association, 2013; Fombonne, 2018), as well as increased awareness and better recognition of borderline cases that were otherwise previously diagnosed as anxiety, bipolar, or other related disorders.

### 3.2 Anxiety in ASDs

Anxiety is characterized by fear. Symptoms can include somatic complaints, such as stomach ache, headache, sleeplessness, and diarrhea, as well as other symptoms including tiredness, irritability, and difficulty concentrating (Beyondblue, 2017). Some level of anxiety is normal. However, when the fear is persistent, excessive and interferes with one's ability to *function normally*, a diagnosis of an anxiety condition may be warranted.

Anxiety symptoms have been noted in individuals with ASD since the disorder was first described more than 70 years ago (Lyons & Fitzgerald, 2007; Uljarević, Nuske, & Vivanti, 2016). Recent research continues to show that those with ASD exhibit significantly higher rates of anxiety symptoms when compared to typically developing individuals (Bellini, 2004; Gadow, Devincent, Pomeroy, & Azizian, 2005; Lopata et al., 2010). Higher rates of anxiety in ASD populations compared to populations with other disorders, including Down's Syndrome, Williams Syndrome, and Conduct Disorder, have also been noted (Evans, Canavera, Kleinpeter, Maccubbin, & Taga, 2005; Green, Gilchrist, Burton, & Cox, 2000; Rodgers, Riby, Janes, Connolly, & McConachie, 2012).

Although the reported rate of anxiety for those with ASD varies widely (e.g., from 13% to 84%), the majority of studies suggest that a realistic estimate is between 40% and 50% (van Steensel, Bögels, & Perrin, 2011).

The majority of studies undertaken exploring anxiety and ASD have focused on very young children, or older adolescents and adults. Fewer studies have been undertaken with school‐aged children, but those studies that have been conducted suggest a high co‐occurrence of anxiety in ASD populations of this age group (Ashburner, Ziviani, & Rodger, 2010; Gjevik, Eldevik, Fjæran‐Granum, & Sponheim, 2011; Lecavalier, 2006). The prevalence of anxiety among school‐aged children is of particular concern considering that anxiety during this period has a negative impact on intellectual functioning and academic achievement, and broadly on a child's overall school‐functioning (Mazzone et al., 2007; Wood, 2006). School may present students with ASD particular cognitive, social and behavioral challenges that may increase levels of anxiety, and conversely, increased anxiety can impair school‐functioning. In addition, teachers tend to perceive students with ASD as having more difficulty with academic success and with anxiety than their typically developing peers (Ashburner et al., 2010). Additional studies of children with an ASD have shown that anxiety negatively impacts a child's ability to participate in home, school, and community settings, and effects child and family well‐being and quality of life above and beyond the core symptoms of ASD (Davis, White, & Ollendick, 2014; Pellecchia et al., 2016). Anxiety also has long term impacts. If left untreated, anxiety persists into adulthood and can progress into other disorders, such as depression (Seligman & Ollendick, 1998; US Public Health Service, 2000). Moreover, chronic anxiety is related to reduced employment opportunities and social networks, and thus is associated with the societal and economic burden (Davis, Ollendick, & Nebel‐Schwalm, 2008; Velting, Setzer, & Albano, 2004).

While it may sometimes be difficult to distinguish between the characteristics of ASD and the characteristics of anxiety, this review assumes that a change in anxiety levels as indicated by changes in standardized and validated measures of anxiety while the diagnosis of ASD remains can be taken as an indicator of a treatment effect on anxiety.

### 3.3 The intervention

Interventions and programs that aim to address anxiety and the challenges that school‐aged children with ASD face in educational environments, may improve their overall school‐functioning and later life outcomes. Against this background, the need for accurate treatment of anxiety in school‐aged children with ASD is evident. There are numerous interventions currently available for the treatment of anxiety in children and young people.

The focus of this review is on interventions designed to help a child's functioning in real‐world settings such as school and the home, although treatment or interventions may be located in a range of settings, including schools, the home, online, and research and support centers. Thus, studies assessing only the impact of pharmacological interventions were excluded, while a study investigating the impact of CBT on academic performance would be included. Research indicates CBT is useful for treating anxiety disorders, but less is known about its efficacy in treating anxiety within ASD populations (Nadeau et al., 2011).

### 3.4 How the intervention might work

Rotheram‐Borus, Swendeman, and Chorpita (2012) proposed that all existing interventions for anxiety incorporate one or more of the following seven elements: (a) psychoeducation, (b) exposure, (c) cognitive restructuring, (d) parent training or parent psychoeducation, (e) relaxation, (f) modeling, and (g) self‐monitoring.

CBT is a relatively popular alternative to pharmacological intervention for anxiety symptoms that incorporates a number of these elements. At its core, CBT involves, as the name suggests, *cognitions* or thoughts and how these may contribute to or alleviate anxiety, and *behavior* or how a person might behave or respond to a situation or experience that may trigger anxiety, as well as how these cognitions and behavior interact. A CBT‐based intervention for young people with ASD and anxiety will probably include educational sessions for the young people, and possibly their parents, about negative thought patterns and cognitive distortions such as “catastrophising” and how these contribute to anxiety (psychoeducation and parent psychoeducation/training) and how to challenge these thought patterns (cognitive restructuring). These sessions might also be combined with other types of intervention like supported exposure to situations that the young people have previously found anxiety‐provoking, such as social interactions, with coaching sessions on how to monitor their thoughts, and to recognize and control physical reactions to stress and anxiety (self‐monitoring and relaxation).

Previous research has indicated that CBT can be effective and efficient in treating anxiety in children and youth as well as adult populations (Kaczkurkin & Foa, 2015; Kendall & Southam‐Gerow, 1996; Otte, 2011), but the core features of ASDs must be considered when determining whether and how the treatment might be appropriate for use with ASD populations. Some characteristics of CBT, such as its highly‐structured, pragmatic focus on current problems may align with features of ASD such as increased need for structure and order, while other aspects such as reliance on verbal communication with the therapist, insight in one's own thoughts, feeling and actions, and recognition of emotions in oneself and others, may prove challenging for some clients with ASD. For these reasons, many CBT‐based treatments for anxiety have been modified specifically for use with ASD populations, including such considerations as replacing group sessions with one‐on‐one treatments sessions, increasing the amount of time dedicated to engagement with the therapist, increasing the number of sessions dedicated to emotion recognition training, adapting activities, and worksheets to the specific strengths and weaknesses of the clients or incorporating clients’ special interests into treatment where appropriate (NICE, 2013).

### 3.5 Why it is important to do the review

Since children spend a significant portion of their day at school, teachers and clinicians working in the education sector have significant responsibility for recognizing signs of ASD and anxiety, and in implementing interventions and supports that are evidence‐based and tailored to the needs of the child. Further, decision making regarding treatment should be informed by the latest evidence available.

A preliminary search of PROSPERO, MEDLINE, the Cochrane Database of Systematic Reviews (apart from this copublished protocol), and the JBI Database of Systematic Reviews and Implementation Reports was conducted and no current or underway systematic reviews on the topic were identified. A number of reviews on various aspects of anxiety in ASD published in the last 10 years were found. These reviews covered phenomenology and prevalence of anxiety (MacNeil, Lopes, & Minnes, 2009; van Steensel et al., 2011; White, Oswald, Ollendick, & Scahill, 2009; Wigham & McConachie, 2014), assessment (Lecavalier et al., 2014; Wigham & McConachie, 2014), and treatment (Johnco & Storch, 2015; Kreslins, Robertson, & Melville, 2015; Sukhodolsky, Bloch, Panza, & Reichow, 2013; Ung, Selles, Small, & Storch, 2015; Vasa et al., 2016). However, none of the reviews published thus far have: (a) focused specifically on school‐aged children with ASD; (b) covered the range of available treatments, but instead focused only on specific treatments, such as, for example, CBT or psychosocial treatments; (c) explored mediators and moderators of treatment outcomes; and (d) provided practical guidance for education professionals and parents to enable increased use of evidence‐based treatments in their everyday practice.

Accordingly, this review aimed to synthesize evidence about interventions to reduce anxiety symptoms in school‐aged children with ASD. While clinical studies were not excluded per se, this review sought to move beyond interventions that were relevant only for clinical practice and care in clinical settings, and prioritized studies that drew out implications for school‐aged children that would help their functioning in real‐world settings such as school and the home. To achieve this aim, the review employed a quantitative (experimental and quasi‐experimental) approach, in order to establish evidence of impact (Joanna Briggs Institute, 2014).

## 4 OBJECTIVES

### 4.1 The problem

The sheer volume of published research, and the different aims, foci, and methodology of those studies, makes evidence‐based practice difficult for professionals, including for those working in the education sector. The current review contributes to providing consolidated sources of information for professionals. Results of the review are intended to inform professionals working in the education sector and parents, but may also inform policymakers in this sector.

Hence, this review aimed to address the following research question.

1. What is the relative effectiveness of interventions for managing anxiety of school‐aged children with ASD that have been used in school, family, and clinical settings?

In the process, this review also identified the following:

- The interventions used for managing anxiety of school‐aged children with ASD in school, family, and clinical settings.
- The evidence‐based practices that school staff, parents, and other professionals can employ to mitigate anxiety‐related symptoms in school‐aged children with ASD.

## 5 METHODS

### 5.1 Criteria for considering studies for this review

#### 5.1.1 Types of studies

While the original strategy did not set limits on the types of studies to be reviewed, the results of initial searching proved so prolific that it was decided, on the basis of quality, to focus on two main types of quantitative studies—RCTs and quasi‐experimental studies (in which a control group was employed but allocation was not strictly randomized). The mixed methods strategy proposed initially in the protocol (Lietz et al., 2018) was thus replaced with a purely quantitative review and meta‐analysis. Otherwise, this review followed the approaches to search strategies and analyses specified in the study protocol (Lietz et al., 2018) which was published by the Campbell Collaboration prior to starting the research.

The studies could occur in schools or out‐of‐school settings (e.g., home, larger community) or clinical settings, as long as the intervention was designed to improve outcomes in real‐world settings.

The comparison groups used in the majority of included studies were waitlist control groups or standard treatment/treatment‐as‐usual (TAU) groups. Two studies, namely vanSteensel_2015^1^ and Ohan_2016, were included as pre‐ and posttest comparisons only. In vanSteensel_2015, the intended comparison group was children with an anxiety disorder but no ASD. Ohan_2016 combined their immediate treatment and waitlist groups after initial testing indicated that there was no significant change in the scores of the waitlist group, thereafter reporting pretreatment and posttreatment scores for the combined group. A third study, Pryor_2016, used a crossover design so only results collected after the first intervention round were used in the current analyses.

#### 5.1.2 Types of participants

The target population for the review is mainstream school‐aged children, diagnosed with ASD (inclusive of autism, ASD, Autistic Disorder, Asperger's Disorder, Asperger Syndrome, atypical autism, PDD‐NOS) by a professional eligible to diagnose these conditions, *and also* experiencing anxiety symptoms or a diagnosis of an anxiety disorder provided by a professional eligible to diagnose such conditions. The majority of included studies (21 of 24) used a screening instrument to confirm the existence of clinically significant levels of anxiety at intake, while the remaining studies relied on parent or teacher reports of elevated anxiety.

If studies included a sample of children in the target population as well as other children (e.g., the general population) and the findings were separated for the ASD subgroup, the study was included in the review whereby the type of ASD diagnosed did not matter. In contrast, if the study findings were not reported separately (e.g., the results for children with ASD and ADHD were combined for analysis), the study was excluded from the review as the impact of the intervention on only the ASD sample would be impossible to isolate.

To be included in the review, either all participants in a study had to be of mainstream school age or a majority of participants had to be of mainstream school age. This meant that while most studies involved young people aged 6–16 years, one study (Piravej_2009) included some younger children (minimum 3 years old) and six studies included slightly older participants (MacKinnon_2014, Pryor_2016, White_2013: max. 17 years; Murphy_2017, van Steensel_2015: max. 18 years; and Hepburn_2019: max. 19 years). No restrictions were imposed in terms of background variables such as socioeconomic status, or profiles of children with ASD with respect to characteristics such as level of cognitive functioning or ASD severity/classification, for example. However, given the types of interventions that were included and the requirement in some studies for the participating children and adolescents to report on their posttreatment anxiety levels, the majority of studies did include some requirements for minimum IQ or at least verbal IQ (VIQ) (generally a full scale or VIQ of 70), and behavioral standards (e.g., exclusion of violent subjects).

#### 5.1.3 Types of interventions

This review included all treatments for anxiety where the large majority of participants were of mainstream school age, with ASD which occurred in schools, families or in clinical settings and that encompassed *at least one* of the elements outlined by Rotheram‐Borus et al. (2012). As such, studies that focused solely on pharmacological interventions (e.g., selective serotonin reuptake inhibitors) were excluded from the review. Given the focus on psychoeducation and cognitive restructuring in Rotheram‐Borus et al.'s (2012) recommendations, many of the included studies used a form of CBT. Those that did not examine CBT encompassed elements of relaxation, modeling, and self‐monitoring.

Two examples of included studies identified by the initial search criteria are provided for illustrative purposes.

Chalfant, A. M., Rapee, R., & Carroll, L. (2006). Treating anxiety disorders in children with high functioning autism spectrum disorders: A controlled trial. *Journal of Autism and Developmental Disorders*, 37 (10), 1842‐1857.

A family‐based, cognitive behavioral treatment for anxiety in 47 children with comorbid anxiety disorders and High Functioning Autism Spectrum Disorder (HFA) was evaluated. Treatment involved 12 weekly group sessions and was compared with a waiting list condition. Changes between pre‐ and post‐treatment were examined using clinical interviews as well as child‐, parent‐ and teacher‐report measures. Following treatment, 71.4% of the treated participants no longer fulfilled diagnostic criteria for an anxiety disorder. Comparisons between the two conditions indicated significant reductions in anxiety symptoms as measured by self‐report, parent report and teacher report. Discussion focuses on the implications for the use of cognitive behavior therapy with HFA children, for theory of mind research and for further research on the treatment components.

Wood, J. J., Drahota, A., Sze, K., Har, K., Chiu, A., & Langer, D. A. (2009). Cognitive behavioral therapy for anxiety in children with autism spectrum disorders: A randomized, controlled trial. *The Journal of Child Psychology and Psychiatry*, 50 (3), 224–234.

Background: Children with autism spectrum disorders often present with comorbid anxiety disorders that cause significant functional impairment. This study tested a modular cognitive behavioral therapy (CBT) program for children with this profile. A standard CBT program was augmented with multiple treatment components designed to accommodate or remediate the social and adaptive skill deficits of children with ASD that could pose barriers to anxiety reduction. Method: Forty children (7‐11 years old) were randomly assigned to 16 sessions of CBT or a 3‐month waitlist (36 completed treatment or waitlist). Therapists worked with individual families. The CBT model emphasized behavioral experimentation, parent‐training, and school consultation. Independent evaluators blind to treatment condition conducted structured diagnostic interviews and parents and children completed anxiety symptom checklists at baseline and posttreatment/postwaitlist. Results: In intent‐to‐treat analyses, 78.5% of the CBT group met Clinical Global Impressions‐Improvement scale criteria for positive treatment response at posttreatment, as compared to only 8.7% of the waitlist group. CBT also outperformed the waitlist on diagnostic outcomes and parent reports of child anxiety, but not children's self‐reports. Treatment gains were maintained at 3‐month follow‐up. Conclusions: The CBT manual employed in this study is one of the first adaptations of an evidence‐based treatment for children with autism spectrum disorders. Remission of anxiety disorders appears to be an achievable goal among high‐functioning children with autism.

The following is an example of study that was excluded due to it being a pharmacological only treatment:

Couturier, J., & Nicolson, R. (2002). A retrospective assessment of citalopram in children and adolescents with pervasive developmental disorders. *Journal of Child and Adolescent Psychopharmacology*, 12(3), 243–248.

Although selective serotonin reuptake inhibitors have been used to treat symptoms of aggression and anxiety in children and adolescents with pervasive developmental disorders (PDDs), there are no published reports of the use of citalopram in this population. The purpose of this study was to examine the benefits and adverse effects of citalopram in a group of children and adolescents with PDDs. Target behaviors included aggression, anxiety, stereotypies, and preoccupations. Seventeen patients with PDDs (14 with autistic disorder, three with Asperger's disorder) (mean age = 9.4 ± 2.9 years; range 4‐15 years) were treated with citalopram for at least 2 months (mean duration of treatment =  7.4 ± 5.3 months; range 1‐15 months). Treatment was initiated at a low dose (5 mg daily) and was increased by 5 mg weekly as tolerated and as necessary. The mean final dose was 19.7  ± 7.8 mg (range 5‐40 mg). Outcome was based on a consensus between clinician and parents, using the Improvement item of the Clinical Global Impressions Scale as a guide. Ten (59%) children were judged to be much improved or very much improved regarding target behaviors. Core symptoms of PDDs (social interactions, communication) did not show clinically significant improvement. Citalopram was generally well tolerated, although four patients developed treatment‐limiting adverse effects: two with increased agitation, one with insomnia, and one with possible tics. The results of this case series suggest that citalopram has beneficial effects on some interfering behaviors associated with PDDs with few adverse effects. Controlled trials are warranted.

Other excluded studies are summarized in Table 1.

**Table 1:** Characteristics of studies excluded at abstract screening stage

Author and Publication Date	Title	Reason for exclusion
Schohl et al. (2014)	A replication and extension of the PEERS intervention: Examining effects on social skills and social anxiety in adolescents with autism spectrum disorders.	Different intervention focus, no anxiety diagnosis or measurement at intake
Ooi et al. (2008)	Effects of cognitive‐behavioral therapy on anxiety for children with high‐functioning autistic spectrum disorders.	Observational study (pre‐post design only, no control group)
Ehrenreich‐May et al. (2014)	An open trial of cognitive behavioral therapy for anxiety disorders in early adolescents with autism spectrum disorders.	Observational study (no control group)

#### 5.1.4 Types of outcome measures

The primary outcome for included studies was anxiety, thus studies that focused on social skills interventions or other symptomatology of ASD as primary outcomes were excluded from this review. The measurement of anxiety (and related terms) had to be undertaken using valid and reliable approaches such as diagnostic interviews, screening instruments, observational ratings, and behavioral checklists—irrespective of the informant (e.g., student, parent, teacher).

Only the immediate posttreatment outcome is included in the current review, as the variety of follow‐up schedules in the studies proved quite large, with 10 studies having no follow‐up, 6 studies following‐up less than 3 months after the end of the intervention, 4 studies after exactly 3 months, and 4 studies more than 3 months after the intervention (see Table 2). Compiling the results from different studies into ranges may have resulted in a loss of data integrity.

**Table 2:** Overview of the studies included in the review

Author and year	Full sample size (M, F)	Age span (mean, *SD*)	Clinical anxiety screening?	IQ screening?	IQ (mean, *SD*)	Comparison	Outcome measures	Intervention type	Individual or group sessions?	Family involvement in sessions?	Delivery format	Subsequent follow‐up
Chalfant, Rapee, and Carroll (2007)	47 (35, 12)	8–13 years (10.8, 1.4)	Yes, ADIS	No, referral documentation for age appropriate language skills		IT v WLC/TAU	RCMAS	CBT: Cool Kids with modification for HFA	Group	Yes	Face to face	None
					SCAS‐P							
					SCAS‐C							
Clarke, Hill, and Charman (2017)	28 (28, 0)	11–14 years (12.7, 0.8)	No, teacher report	Yes, WASI	IT: 97.71 (11.37)	IT v WLC/TAU	SCAS‐P	CBT: Exploring Feelings	Group	No	Face to face	6/8 weeks
					WLC: 106.57 (13.93)		SCAS‐C					
Conaughton, Donovan, and March (2017)	42 (36, 6)	8–12 years (9.7, 1.3)	Yes, ADIS	No, required to read and write English at 8‐year‐old level		IT v WLC/TAU	SCAS‐P	CBT: BRAVE Online	Individual	No	Computer (online)	3 months
					SCAS‐C							
Corbett, Blain, Ioannou, and Balser (2017)	30 (24, 6)	8–14 years (11.0, 2.2)	No	Yes, WASI (IQ ≥ 70)	IT: 106.06 (16.83)	IT v WLC/TAU	STAI‐C	Social Emotional Neuro Science Endocrinology (SENSE) Theater therapy	Group	No	Face to face	None
					WLC: 95.85 (21.19)							
Fujii et al. (2013)	12 (9, 3)	7–11 years (8.8, 1.6)	Yes, ADIS	No, exclusion criteria of IQ < 70		IT v WL/TAU	ADIS‐CRS	CBT: Building Confidence with modification for ASD	Individual	Yes	Face to face	None
Hepburn, Blakeley‐Smith, Wolff, and Reaven (2016)	33 (27, 6)	7–19 years (11.8, 2.3)	Yes, SCARED	No	categories only reported	IT v Comparison (not strictly WL as recruited later)	SCARED‐P	CBT: Facing Your Fears Telehealth delivery (videoconferencing)	Group	Yes	Video conference	None
Luxford, Hadwin, and Kovsoff (2017)	35 (31, 4)	11–15 years (13.2, 1.1)	Yes, SAS or Spence	Yes, WASI (FIQ and VIQ ≥ 70)	IT: 105.44 (17.83)	IT v WLC/TAU	SCAS‐C	CBT: Exploring feelings	Group	No	Face to face	6 weeks
					WLC: 102 (11.30)		SCAS‐P					
							SWQ‐C					
MacKinnon, Comerford, Parham, and Roberts (2014)	24 (20, 4)	9–17 years (12.4, 1.8)	Yes, ADIS	No	4 participants described as having “mild ID,” no further information	IT v WLC/TAU	ADIS‐P	CBT: Cool Kids with modifications for ASD/ID	Group	Yes	Face to face	None
							SCAS‐P					
							SCAS‐C					
McConachie et al. (2014)	32 (28, 4)	9–13 years (11.8, 1.4)	Yes, ADIS	Yes, WAI (FIQ > 69)	T: 100.5 (17.2)	IT v WL/TAU	ADIS‐C	CBT: Exploring Feelings, inclusion of additional introductory session	Group	Yes	Face to face	6 months and 9 months
					WLC: 100.7 (12.0)		SCAS‐P					
							SCAS‐C					
McNally Keehn, Lincoln, Brown, and Chavira (2013)	22 (21, 1)	8–14 years (11.3, 1.5)	Yes, ADIS	Yes, WASI (FIQ ≥ 70)	IT: 108.42 (17.7)	IT v WLC/TAU	ADIS‐P	CBT: Coping Cat, with modifications for ASD	Group	Yes	Face to face	2 months
					WLC: 110.40 (17.39)		SCAS‐P					
							SCAS‐C					
Murphy et al. (2017)	36 (22, 14)	12–18 years (15.3, 1.8)	Yes, ADIS	No, required main‐stream school attend‐ance (de facto IQ > 7)	Not reported	CBT v alt treatment (counseling)	ADIS‐CRS	CBT: Multimodal Anxiety and Social Skills Intervention for adolescents with ASD (MASSI)	Group(+ some individual)	No	Face to face	3 months
							CASI‐Anx					
Ohan et al. (2016)	24 (18, 6)	8–12 years (9.1, 1.2)	Yes, SCARED	No, exclusion of comorbid ID based on family report	Not reported	IT v WLC/TAU	CALIS‐P	CBT: Cool Kids adaptation for ASD, further adapted to reduce number of sessions to fit with school term	Group	Yes	Face to face	None
							CALIS‐C					
							SCAS‐C					
							SCAS‐P					
Piravej, Tangtrongchitr, Chandarasiri, Paothong, and Sukprasong (2009)	60 (49, 11)	3–10 years (4.7, 1.8)	No	No	Not reported	TTM + SI v SI	CPRS	Thai traditional massage	Individual	No (presence only)	Face to face	None
Pryor (2016)	24 (22, 2)	7–17 years (12.3, 2.4)	Yes, ADIS	Yes, WASI (VIQ ≥ 70)	Group 1:	CCAL v social skills intervention (TSE), cross‐over	ADIS‐P	CBT: CCAL, computer‐assisted	Individual	No	Computer	None
					FIQ: 91.67 (12.32); VIQ: 88.75 (11.83)		MASC‐C					
					Group 2		MASC‐P					
					FIQ: 102. 58 (12.67);		SCAS‐C					
					VIQ: 100.33 (11.50)		SCAS‐P					
Reaven et al. (2009)	33 (26, 7)	8–14 years (11.8, 1.9)	Yes, SCARED	Yes, WASI FIQ ≥ 70, initial VIQ ≥ 80 but revised to “spontaneous, functional verbal language of the complexity required to complete Module III of the ADOS (Autism Diagnostic Observation Schedule).” If WISC‐IV conducted within past 2 years, WASI not performed	Total sample: FIQ: 102.46 (16.22); VIQ: 102.65 (19.51); Non‐VIQ: 101.76 (15.07). No group comparison presented.	IT v WLC/TAU	SCARED‐C	CBT: manual	Group	Yes	Face to face	None
							SCARED‐P					

Reaven, Blakeley‐Smith, Culhane‐Shelburne, and Hepburn (2012)	50 (48, 2)	7–14 years (10.4, 1.7)	Yes, SCARED	Yes, WASI (if not assessed in past 3 years) initial VIQ ≥ 80 but subjects with VIQ < 80 evaluated for participation and 3 included	IT: FIQ: 107.80 916.85); VIQ: 107.00 (19.51); Non‐VIQ: 109.67 (16.38). TAU: FIQ: 102.23 (17.33); VIQ: 100.73 (18.98); Non‐VIQ: 105.04 (17.86).	IT v WLC/TAU	ADIS‐P	CBT: Facing Your Fears	Group	Yes	Face to face	3 months and 6 months

Sofronoff, Attwood, and Hinton (2005)	71 (62, 9)	10–12 years (10.6, 1.1)	Yes, SCAS and SCAS‐P. SCAS dropped as outcome measure	No requirements for inclusion stated, short‐form WISC‐III IQs reported	IT child only:	IT child only v IT parent + child v WLC/TAU	SCAS‐P	CBT: brief	Group	No (Group 1)	Face to face	6 weeks
					FIQ: 107.5 (27.3)					Yes (Group 2)		
					IT parent + child:							
					FIQ: 105.6 (21.2)							
					WLC: FIQ: 101.0 (27.2)							
Storch et al. (2013)	45 (36, 9)	7–11 years (8.9, 1.3)	Yes, ADIS	No, exclusion criteria of FIQ or VIQ < 70 (parental report during enrollment)	Not reported	IT v WLC/TAU	ADIS‐CRS	CBT: Behavioral Interventions for Anxiety in Children with Autism (BIACA)	Individual	Yes	Face to face	3 months
							MASC‐P					
							PARS					
							RCMAS					
Storch et al. (2015)	31 (25, 6)	11–16 years (12.7, 1.3)	Yes, ADIS‐IV‐C/P	No, exclusion criteria of WASI IQ < 80 (parental report during enrollment, no report of conducting screening)	Not reported	IT v TAU	ADIS‐CRS	CBT: BIACA modified for adolescents	Individual	Yes	Face to face	1 month
							MASC‐P					
							PARS					
							RCADS					
Sung et al. (2011)	70 (66, 4)	9–16 years (11.2, 1.8)	Yes, SCAS‐C	Yes, WISC‐IV Verbal Comprehension ≥ 80 and Perceptual Reasoning ≥ 90	CBT: Verbal Comp: 100.25 (13.97)* sig diff to SR group; Perceptual Reasoning: 108.00 (12.26)	CBT v social recreation program	SCAS‐C	CBT: elements of Coping Cat, Exploring Feelings, and unpublished programs, modified for Asian cultural context	Group	No	Face to face	3 months and 6 months
					SR: Verbal Comp: 93.06 (12.81); Perceptual Reasoning: 105.94 (11.07)							
van Steensel and Bögels (2015)	79 (58, 21)	7–18 years (11.8, 2.7)	Yes, ADIS‐C/P	No, inclusion criteria of ≥70 stated but estimated based on school progression (in case of poor school performance, IQ was assessed but found to still be >70)	Not reported	CBT ASD + AD v AD only, ASD + AD pre‐post	ADIS‐CRS	CBT: Discussing + Doing = Daring	Individual	Yes	Face to face	3 months, 1 year and 2 years
							SCARED‐C					
							SCARED‐P					
White et al. (2013)	30 (23, 7)	12–17 years (14.6, 1.5)	Yes, ADIS‐C/P	Yes, inclusion criteria of current VIQ ≥ 70 and no previous diagnosis of ID (WASI subscales of Vocabulary and Similarities conducted as confirmation)	IT:	IT v WLC/TAU	CASI‐Anx	CBT: MASSI	Group (+ some individual)	Yes	Face to face	None
					VIQ: 100.07 (16.49)		PARS					
					WLC:							
					VIQ: 94.07 (11.92)							
Wood et al. (2009)	40 (27, 13)	7–11 years (9.2, 1.5)	Yes, ADIS‐C/P	Exclusion criteria of VIQ < 70 (previous assessment or WISC/IV at intake)	Not reported	IT v WLC/TAU	ADIS‐CRS	CBT: Building Confidence with modifications	Individual	Yes	Face to face	3 months
							MASC‐P					
							MASC‐C					
Wood et al. (2015)	33 (23, 10)	11–15 years (12.3, 1.1)	Yes, ADIS‐IV‐C/P	Inclusion criteria on of WISC‐IV FIQ ≥ 85, evaluated if no current (3 years) documentation	Not reported	IT v WLC/TAU	ADIS‐CRS	CBT: BIACA	Individual	Yes	Face to face	1 month
							MASC‐P					
							PARS					
							RCADS					

Abbreviations: ADIS, Anxiety Disorders Interview Schedule; ASD, autism spectrum disorder; CASI‐Anx, Childhood Anxiety Sensitivity Index; CALIS, Child Anxiety Life Interference Scale; CBT, cognitive behavioral therapy; CCAL, Camp Cope‐A‐Lot; CPRS, Conners’ Parent Rating Scales; FIQ, full scale IQ; IT, intervention group; MASC, Multidimensional Anxiety Scale for Children; PARS, Pediatric Anxiety Rating Scale; RCADS, Revised Child Anxiety and Depression Scale; RCMAS, Revised Children's Manifest Anxiety Scale; SCARED, Screen for Child Anxiety Related Disorders; SCAS, Spence Child Anxiety Scale; STAI‐C, State‐Trait Anxiety Inventory for Children; SWQ, Social Worries Questionnaire; TAU, treatment as usual; VIQ, verbal IQ; WASI, Wechsler Abbreviated Scale of Intelligence; WISC, Wechsler Intelligence Scale for Children; WLC, wait‐list control.

#### 5.1.5 Types of settings

The settings in which the intervention was applied were real‐world settings such as school or home. While 19 of the interventions were conducted in a clinical setting (either a university‐based clinic or a community clinic, such as Child and Adolescent Mental Health), the intention of the studies was to address issues that were pertinent to the subjects’ lives—either at home or in school.

### 5.2 Search methods used for the identification of studies

Our search strategy identified published as well as unpublished literature, first, via electronically searching 12 bibliographic databases and, second, by searching additional gray literature sources such as selected websites, repositories, and research registers. We also manually searched targeted journals and reference lists and contacted key researchers in the field of autism to inquire about studies. To ensure our search was as extensive as possible, we balanced our search strategy as far as was practical, toward a sensitive search rather than a precise search.

In summary, studies were included in the review if they met the following criteria:

1. All or the large majority of the patient/client population were mainstream school‐aged young people diagnosed with ASD (inclusive of autism, ASD, Autistic Disorder, Asperger's Disorder, Asperger Syndrome, atypical autism, and PDD‐NOS) by a professional eligible to diagnose these conditions, *and also* experiencing anxiety symptoms or a diagnosis of an anxiety disorder provided by a professional eligible to diagnose such conditions;
2. The intervention was focused on reducing anxiety symptoms and included at least one of the following seven elements: (a) psychoeducation, (b) exposure, (c) cognitive restructuring, (d) parent training or parent psychoeducation, (e) relaxation, (f) modeling, and (g) self‐monitoring.
3. At least one outcome measure was a standardized continuous measure of anxiety (parent, clinician, or self‐reported).
4. The study was published between the years 1996 and 2018.

#### 5.2.1 Electronic searches

A broad range of bibliographic databases were electronically searched for studies that matched our inclusion criteria:

- Academic Search Complete (via EBSCO)
- A+ Education (via Informit)
- British Education Index (via EBSCO)
- CBCA Complete (via Proquest)
- CINAHL (via EBSCO)
- Education Research Complete (via EBSCO)
- EMBASE (via Elsevier)
- ERIC (via EBSCO)
- PsycINFO (EBSCO)
- PubMed
- SCOPUS (via Elsevier)
- SocINDEX (via EBSCO)

Our general search statement, set out below, was customized to fit the available search features of the bibliographic databases (see Appendix A in the Supporting Information Material for the customized statements). In this general search statement, the * symbol was used to indicate where our search covered variations in the root of the word.

(ASD *OR* Asperger* *OR* autis* *OR* Pervasive Developmental Disorder *OR* PDD NOS *OR* PDD unspecified) *AND* (Anxiety *OR* anxious *OR* internali* *OR* fear) *AND* (Student *OR* child* *OR* adolescen* *OR* preadolescen* *OR* pre adolescen* *OR* youth *OR* teen* *OR* teen age* *OR* young people *OR* young person *OR* boy *OR* girl) *AND* (Intervention *OR* treatment *OR* therap* *OR* psychotherap* *OR* evaluation *OR* outcome *OR* program *OR* trial* *OR* experimental *OR* control group *OR* random* *OR* best practi* or evidence based)

#### 5.2.2 Searching other resources

Other resources that were search included gray literature, theses, conference proceedings, research reviews, purposely selected websites, reference lists from previously identified articles, and by contacting researchers and colleagues in the field and from the review's advisory group.

*Google* was used to identify gray literature from websites in the government, organization, and education domains (*site:gov, site:edu, site:org)*. References were checked up to the first 200 results. The following search statement was used within the limits of the three specified domains was:

(autism *OR* autistic *OR ASD OR* asperger *OR* "PDD NOS" *OR* "PDD unspecified" *OR* "pervasive developmental") *AND* (anxiety *OR* anxious) *AND* (student *OR* child *OR* children *OR* adolescent *OR* youth *OR* teen *OR* boy *OR* girl) *AND* (intervention *OR* treatment *OR* therapy *OR* psychotherapy)‐pubmed filetype:pdf

*OpenGrey* (European) was used to identify relevant European gray literature. The search statement was:

*(*asd *OR* Asperger* *OR* autis* *OR* Pervasive Developmental Disorder* *OR* PDD NOS *OR* PDD unspecified) *AND* (Anxiety *OR* anxious *OR* internali* *OR* fear) *AND* (Student *OR* child* *OR* adolescen* *OR* preadolescen* *OR* pre adolescen* *OR* youth *OR* teen* *OR* teenage* *OR* young people *OR* young person *OR* boy *OR* girl)

*Institutional repositories*: We searched the “Contents” of the Directory of Open Access Repositories (OpenDOAR) to identify research papers from institutional repositories. Our general search statement was customized to fit with the available search fields in the OpenDOAR Google Custom Search. Our search statement was:

(autism *OR* asd *OR* asperger *OR* "PDD NOS" *OR* "PDD unspecified" *OR* "pervasive developmental") *AND* (anxiety *OR* anxious) *AND* (student *OR* child *OR* children *OR* adolescent *OR* youth *OR* teen *OR* boy *OR* girl) *AND* (intervention *OR* treatment *OR* therapy *OR* psychotherapy) ‐pubmed –ncbi

*Theses*: For Networked Digital Library of Theses and Dissertations, a customized search strategy was used and the search limited by available population tags:

(asperger* *OR* autistic *OR* autism *OR* asd) *AND* (anxiety *OR* anxious) *AND* (treatment* *OR* intervention* *OR* therapy *OR* psychotherapy)

For WorldCat, a customized search strategy was used and the search limited by

Thesis/dissertation:

*(*Asperger* *OR* autistic *OR* autism *OR* asd) *AND* (anxiety *OR* anxious) *AND* (intervention* *OR* treatment* *OR* therap* *OR* psychotherapy*)

For American Doctoral Dissertations (EBSCO), the search statement was:

(Asperger* *OR* autism *OR* autistic *OR* ASD *OR* PDD *OR* "Pervasive Developmental") *AND* (anxiety *OR* anxious *OR* fear *OR* internal*)

*Conference proceedings*: In addition to conference proceedings and papers indexed in our selected databases, we identified conference literature via a search on SCOPUS which is a multidisciplinary database. This search was limited to conference papers in the collections other than the Social Sciences, Humanities, or Neuroscience as these collections were covered elsewhere. The search statement was:

TITLE‐ABS‐KEY((asperger* *OR* autis* *OR* asd *OR* "Pervasive Developmental" W/0 disorder* *OR* "PDD NOS" *OR* "PDD unspecified")) *AND* TITLE‐ABS‐KEY((anxiety *OR* anxious *OR* internali* *OR* fear)) *AND* TITLE‐ABS‐KEY((student *OR* child* *OR* adolescen* *OR* preadolescen* *OR* (pre W/0 adolescen*) *OR* youth *OR* teen* *OR* (teen W/0 age*) *OR* "young people" *OR* "young person" *OR* boy *OR* girl)) *AND* TITLE‐ABS‐KEY((intervention *OR* treatment *OR* therap* *OR* psychotherap* *OR* evaluation *OR* outcome *OR* program* *OR* trial* *OR* experimental *OR* (control W/0 group) *OR* random* *OR* (best W/0 practi*) *OR* "evidence based")))

*Research reviews*: Wherever possible, the following search statement was executed in the list of resources below:

(asperger *OR* autism *OR* autistic *OR* ASD *OR* "pervasive developmental" *OR* "PDD NOS")

- Campbell Library
- Cochrane Central Register of Controlled Trials (CENTRAL)

- The JBI Database of Systematic Reviews and Implementation Reports.
- Database of Promoting Health Effectiveness Reviews (DoPHER)
- Evidence for Policy and Practice Information and Coordinating Centre (EPPI‐Centre)
- Cochrane Database of Systematic Reviews
- PROSPERO International prospective register of systematic reviews

Where further refinement was necessary, we added additional search terms relating to the concept of anxiety.

*Targeted searches of selected websites*: We explored the websites of selected agencies, research centers and professional associations including the following:

- Agency for Healthcare Research and Quality
- The Association for Science in Autism
- Australasian Society for Autism Research
- Autism Centre of Excellence—Griffith University
- Autism CRC
- Autism Europe
- Autism Ladder
- Autism‐Open Access
- Autism Program at Yale University
- Autism Research Centre—Cambridge University
- Autism Research Centre
- Autism Research Institute
- Autism Research, Policy, Practice
- Autism Research Trust
- Autism Science Foundation
- Autism Speaks
- Autism Intervention Research Network on Physical Health (AIR‐P)
- Autism Society
- Autism Spectrum Australia (Aspect)
- Autistica
- The Cambridge Autism Research Centre
- Center for Autism Research Excellence (CARE) Boston University
- Center for Autism Research
- Center for Autism Research and Treatment (CART)
- Centers for Disease Control and Prevention – Autism Spectrum Disorder
- Center for Excellence in Autism Research at the University of Pittsburgh
- Child Study Center—Yale School of Medicine
- Global Research in Autism and Neurodevelopment
- Interactive Autism Network
- International Society for Autism Research
- Kennedy Krieger Institute: Autism Spectrum Disorders Research
- Medline Plus Healthline
- National Autism Center
- The National Autistic Society
- National Institute of Health Care Excellence (NICE)
- National Institute of Mental Health
- National Institutes of Health (NIH)
- National Database for Autism Research
- New York Academy of Medicine (NYAM) Gray Literature Report
- Olga Tennison Autism Research Centre, La Trobe University
- Research Autism
- Scottish Autism Research Group
- Simons Foundation Autism Research Initiative
- Vanderbilt Evidence‐based Practice Center
- World Health Organization—Digital Library

*Reference lists*: We searched the reference lists of previously published reviews and meta‐analyses that we identified as well as the reference lists of each of the studies identified for our analysis.

*Current literature*: After our initial search, we set up alerts in Google Scholar and, where possible, in the bibliographic databases, in order to identify any new literature within the time of our study. New table of content alerts were also set up for key journal titles including Research in Autism Spectrum Disorders, Autism, and the Journal of Autism and Developmental Disorders. Alerts were manually scanned for any new references that fitted our search criteria.

*Colleagues*: Contact was also made with researchers and colleagues in the field and from the review's advisory group, to identify any additional studies, particularly those that might have been ongoing or unpublished at the time of our work.

*Ongoing trials*: We identified current and ongoing trials via the following trial registries:

- International Clinical Trials Registry Platform Search Portal
- Clinical Trials.Gov
- Trials Register of Promoting Health Interventions (TRoPHI)
- Cochrane Central Register of Controlled Trials ‐ Cochrane Library

We searched for the following terms:

*(Asperger OR autism OR autistic OR asd OR "pervasive developmental" OR "pdd nos")*

Where further refinement was required, we included anxiety and/or trials limited to children.

#### 5.2.3 Publication date range

Our searches were limited to a publication date range of 1996–2018. We selected 1996 as the earliest publication date in order to narrow the scope of interventions to current approaches used in the last 20 years. Given the development of understanding in this field, we believe that interventions before this date would be less progressive in their approach. The initial database searches were conducted between May 4, 2017, and June 7, 2017. Updates of database searches were conducted via alerts based on the original search statements or by rerunning search statements, up to December 31, 2018.

#### 5.2.4 Other criteria

The searches in our selected sources were not restricted by geography, language, publication type, or by publication status. However, the selected sources are focused on the English language in keeping with our database subscriptions and the primary language of the authors.

### 5.3 Data collection and analysis

#### 5.3.1 Selection of studies

As a first step in the screening process, four reviewers independently assessed titles and abstracts of a purposely heterogeneous subset of five studies identified through the searches. The purpose of this step was twofold: first, it determined their potential eligibility for inclusion in the review and second it served to develop a common understanding and application of inclusion criteria. Once a consensus regarding the application was reached, all abstracts were assessed by at least two reviewers. Where two reviewers disagreed regarding the inclusion of an abstract in the study, resolution was sought through discussion with the full project team. At the end of this step, studies that clearly did not meet the criteria, as well as duplicates, were removed.

#### 5.3.2 Data extraction and management

Full‐text articles were then retrieved for the included abstracts. Reviews of the full‐text articles were undertaken independently by two reviewers (K. H. and K. D.). Any discrepancies were resolved through discussion and, where necessary, further details added to data definitions which may have been unclear. The checklists used during this review phase are presented in Appendices B and C in the Supporting Information Material, along with a summary of the appraisal results for excluded papers.

Once papers were selected for inclusion in the review, data were independently extracted in duplicate by authors K. D. and K. H. using the standardized data extraction tools from JBI SUMARI (see Appendix D in the Supporting Information Material for the quantitative data extraction form, which was operationalized in a spreadsheet). Discrepancies were checked and resolved. The data extracted included specific details about the interventions, populations, study methods, and outcomes of significance to the review question and specific objectives.

#### 5.3.3 Assessment of risk of bias in included studies

The risk of bias assessment was carried out by authors K. H. and K. D. The assessment was informed by initial data extraction in JBI SUMARI and conducted using the Cochrane Collaboration's guidelines for assessing risk of bias (Higgins, Altman, & Sterne, 2011). Risk of bias in the selected studies was rated as high risk (bias that potentially reduces the reliability of the results), or low risk (bias that is unlikely to alter the results), with an unclear category used in cases in which there was insufficient information in the published study for the judgment of bias to be made. As the majority of included studies were RCTs, the risk of bias assessment focused on methodological issues pertaining to this form of study—sequence generation, allocation concealment, blinding of participants and personnel, blinding of outcome assessment, attrition, selective reporting, and other sources of bias. As a consequence, the quasi‐experimental studies that were included in this review received higher ratings of risk of bias, particularly in terms of selection bias.

#### 5.3.4 Measures of treatment effect

Only one outcome per respondent group was used in the quantitative syntheses to avoid double counting. In cases where there was more than one outcome measure per respondent available, we selected an outcome based on assessments of validity published in Wigham and McConachie (2014) and Lecavalier et al. (2014), the frequency of use across the included studies, and the availability of data appropriate for the meta‐analyses.

Separate statistical analyses were conducted based on the informant for the outcome measures—namely parent, clinician, and self/student. While two of the included studies collected anxiety outcome measures from teachers of the subjects (Chalfant_2007; Luxford_2017), there were not enough teacher‐informants to conduct a separate analysis. In addition, the extent of missing data for these measures was enough to raise concerns about reliability.

In accordance with the JBI SUMARI meta‐synthesis program the *SMD*, reported as Cohen's *d*, and its 95% CI was used as the summary estimate of treatment effect size and based on the posttreatment/wait‐list scores reported in each study. This summary statistic was selected as all studies included continuous measures and all measures were in the same direction (i.e., higher scores indicating higher levels of behavior or impact of symptoms) and thus no adjustments were required. In addition to Cohen's *d*—although not available in the JBI SUMARI program at the time of analyses—Hedges’ *g* is another method (i.e., formulae) commonly used for the computation of SMD. Cohen's *d* and Hedges’ *g* differ in that the latter uses the version of the standard deviation formula which divides by N‐1, whereas the former divides by N. While, therefore, Hedge's *g* is often preferred for reviews involving studies with small sample sizes its use would not have led to different conclusions having been drawn from the results of the current review.

#### 5.3.5 Unit of analysis issues

Some of the included studies deviated from standard treatment versus control comparisons, in employing crossover designs (Pryor_2016), inclusion of more than one treatment group (Sofronoff_2005) or inclusion of a control group without ASD who also received treatment (vanSteensal_2015).

For the crossover design, data from the baseline and the end of the first phase (prior to crossover) were used, effectively treating the alternative treatment group as a “TAU” control. Sofronoff_2005 included two treatment arms, one in which children received CBT on their own and another in which their parents participated in the treatment with them. While previous reviews have pooled the results of these two treatment arms (e.g., Kreslins et al., 2015; Ung et al., 2015), we elected to include them separately, as they do represent two different forms of intervention.

The study design of vanSteensal_2015 was relatively more complex, compared to other included studies, in that it included two treatment groups—one with ASD and anxiety disorders, one with anxiety disorders but no ASD diagnosis—and a wait‐list control (WLC) subgroup of the ASD group. While the argument could have been made to exclude this study for a lack of formal diagnosis of ASD for those participants in the “anxiety disorders only” comparison group, it was decided to retain the study and focus on the immediate treatment versus WLC comparison within the ASD group.

#### 5.3.6 Assessment of heterogeneity

Quantitative data were, where possible, pooled by way of statistical meta‐analysis. Weighted mean differences and their 95% CIs were calculated for analysis. Heterogeneity was assessed statistically using the standard *χ* ^2^ and also explored using subgroup analyses based on the different study designs included in this review.

#### 5.3.7 Assessment of reporting biases

Assessing risk of publication bias was an important task because of its potential influence on estimates of intervention effects. This review analyzed possible publication bias by implementing the trim‐and‐fill method (Duval & Tweedie, 2000; Schwarzer, 2007), providing an initial assessment of whether unpublished data on ASD and anxiety interventions (likely to have null results) was evident (Uljarević & Hamilton, 2013).

#### 5.3.8 Data synthesis approach

Separate statistical analyses were carried out for clinician‐reported, parent‐reported, and self‐reported outcome measures of anxiety. Studies were also coded dichotomously for two possible moderator variables: (a) family involvement or student‐only and (b) group or individual format. *SMD* was chosen as the summary estimate of treatment effect, appropriate for the continuous outcome measures that were being analyzed. Often, *SMD* s of 0.2, 0.5, and 0.8 are taken to be indicative of small, moderate, and large effects, respectively (Cohen, 1988). However, heeding concerns by Valentine and Cooper (2003) and Lipsey et al. (2012) that effect in the field of education are likely to be small and risk being overlooked if based on Cohen's interpretation, effect sizes were interpreted in an educational context using the metric developed by Higgins et al. (2013) for the UK Education Endowment Foundation. Accordingly, *SMD*s of 0.05, 0.19, 0.45, and 0.70 were taken to be indicative of low, moderate, high, and very high effects, respectively. Moreover, these can be interpreted, respectively as, 1 month, 3 months, 6 months, and 9 months additional developmental progress. A random effects meta‐analysis was employed due to the variability in outcome measurement instruments and interventions across the included studies.

Using the JBI SUMARI meta‐synthesis program, *SMD* (Cohen's *d*) estimates were calculated based on the posttreatment mean scores and standard deviations provided in each study. Since the direction of the scales was the same for all outcome measures, no adjustments of the scores were required. The statistical significance level was set at *p* < .05. Forest plots were used to illustrate results from individual studies. In the case of multiple treatment arms, such as Sofronoff_2005, the scores of both intervention groups (ITs) were compared to the control group score. Similarly, if a study reported more than one outcome measure for a respondent, then both outcomes have been reported, rather than presenting an average score as previous systematic reviews have done (e.g., Kreslins et al., 2015; Lang, Regester, Lauderdale, Ashbaugh, & Haring, 2010; Perihan et al., 2019; Sukhodolsky et al., 2013; Ung et al., 2015).

#### 5.3.9 Sensitivity analysis

Given the diversity of interventions and the potentially small sample of included studies within each intervention type, it was important to conduct a sensitivity analysis of the impact of a single study, particularly if it is an outlier, on the overall observed effect size for interventions in any meta‐analysis. The main sensitivity analysis conducted, focused on excluding single studies which may have had an unduly large effect on the results. Results were then compared to provide an indication of the robustness of the review's findings.

## 6 RESULTS

### 6.1 Description of studies

#### 6.1.1 Results of the search

The search of the databases yielded 3,417 records, with an additional 177 records identified through other sources. Removal of duplicates resulted in a total of 2,337 records, which were screened based on the title and abstract by three of the authors, with 2,218 records being excluded.

**Figure 1:**
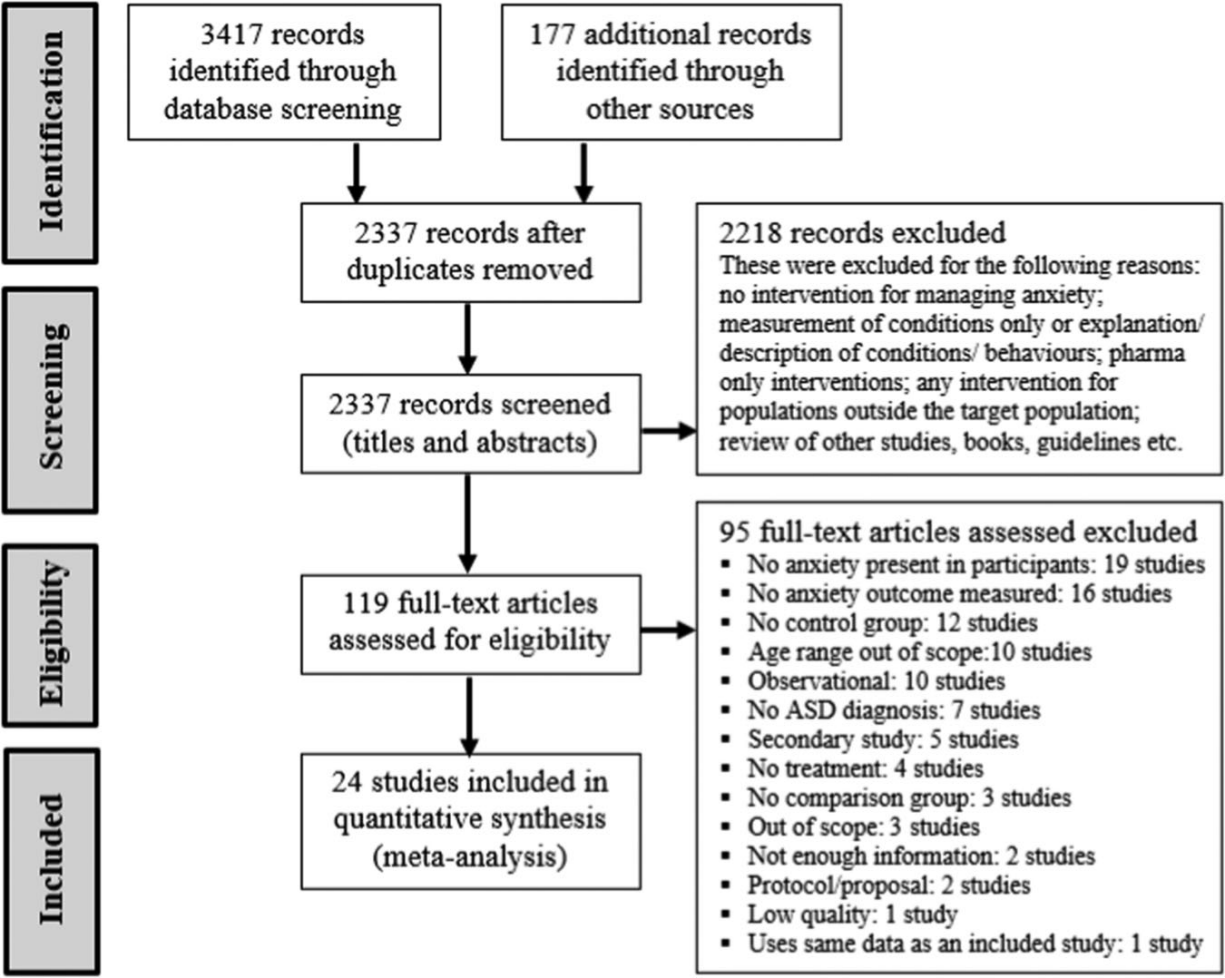
PRISMA flow diagram outlining process of study selection

The remaining 119 full‐text articles and theses were assessed for eligibility and 94 excluded as not meeting the search criteria. One further study was subsequently excluded as a reanalysis of data reported in an already included study. A total of 24 studies were thus included in the quantitative meta‐synthesis. A flow diagram of the study selection is presented in Figure 1.

#### 6.1.2 Included studies

Twenty‐four studies examining interventions for anxiety for children and adolescents with ASD are included in this review, identified by first author and publication date: Chalfant_2007; Clarke_2017; Conaughton_2017; Corbett_2017; Fujii_2013; Hepburn_2016; Luxford_2017; MacKinnon_2014; McConachie_2014; McNally‐Keehn _2013; Murphy_2017; Ohan_2016; Piravej_2009; Pryor_2016; Reaven_2009; Reaven_2012; Sofronoff_2005; Storch_2013; Storch_2015; Sung_2011; vanSteensel_2015; White_2013; Wood_2009; Wood_2015.

#### 6.1.3 Study location

Five of the studies were conducted in Australia, 12 in the United States, 4 in the United Kingdom (England), and 1 each in Singapore, Thailand, and The Netherlands. Twenty of the studies were set in clinical settings attached to medical or university institutions, while two were home‐based interventions and two were school‐based.

#### 6.1.4 Study design

Six of the studies were classified as quasi‐experimental designs, in that they employed a control or comparison group that was not randomly assigned, while the remaining 18 studies used a randomized WLC design (with varying degrees of fidelity).

#### 6.1.5 Participants

A total of 931 (764 male and 167 females) participants most of whom were of mainstream school age (6–16) were included in these studies (note that subjects in pre‐post studies only counted once). Six of the studies included older adolescents (up to 19 years of age) while one study included children as young as 3 years old. All studies had inclusion criteria of a documented diagnosis of ASD (often confirmed during intake assessments) as well as either a concurrent diagnosis of an anxiety disorder (again, confirmed during intake) or reports from parents or educators of significant levels of anxiety.

The majority of the studies (21 of 24) limited participants to individuals with functioning above a certain level of cognitive ability, most commonly a full scale or verbal IQ of 70 or above, or in one case, the ability to read and write (in English) at an 8‐year‐old level as a minimum. Only three studies did not place restrictions (either explicitly or de facto by requiring that participants be attending a mainstream school) on the cognitive functioning of participants (Hepburn_2016; MacKinnon_2014; Piravej_2009). Further information, where available, on the cognitive functioning of participants in each study, is presented in Table 2.

#### 6.1.6 Interventions

Twenty‐two of the studies used a CBT intervention, with some developed specifically for use with participants with ASD (e.g., Exploring Feelings; BIACA and MASSI). Of these, 15 studies involved interventions that included parental involvement (Chalfant_2007; Fuji_2013; Hepburn_2016; MacKinnon_2014; McConachie_2014; McNally‐Keehn_2013; Ohan_2016; Reaven_2009; Reaven_2012; Storch_2013; Storch_2015; van Steensel_2015; White_2013; Wood_2009, Wood_2015), and six studies involved student‐only CBT treatments (Clarke_2017; Conaughton_2017; Luxford_2017; Murphy_2017; Pryor_2016; Sung_2011). One study (Sofronoff_2005) had two treatment arms, with and without parental involvement.

Rather than a CBT intervention, Corbett_2017 used peer‐mediated theater therapy in a group context to address social anxiety by building the social‐emotional skills of participating adolescents, while Piravej_2009 examined the benefits of Thai traditional massage in a one‐on‐one context to internalizing, externalizing, and sleep behaviors compared to the standard sensory integration treatment available to participants.

In development since previous reviews of treatments for anxiety in the ASD population, three studies examined the effectiveness of CBT treatments (either published or newly developed) designed for computer delivery (Conaughton_2017; Pryor_2016) or videoconferencing (Hepburn_2016).

#### 6.1.7 Outcome measures

Across the 24 studies, 12 outcome measures of anxiety were used to varying extent and with different respondents (clinician, parent, child). Most of these measures are standardized, validated measures of anxiety for use by clinicians (e.g., psychologists). The measures, as indicated in Table 2, were the: Anxiety Disorders Interview Schedule (ADIS: Silverman & Albano, 1996); the Child Anxiety Life Interference Scale (CALIS: Lyneham et al., 2013); the Childhood Anxiety Sensitivity Index ‐ Anxiety (CASI‐Anx: Sukhodolsky et al., 2008); Conners’ Parent Rating Scales—Anxiety (CPRS: Conners, 1989); the Multidimensional Anxiety Scale for Children (MASC: March, Parker, Sullivan, Stallings, & Conners, 1997); the Pediatric Anxiety Rating Scale (PARS: RUPP Anxiety Study Group, 2002); the Revised Child Anxiety and Depression Scales (RCADS: Chorpita, Moffitt, & Gray, 2005); the Revised Children's Manifest Anxiety Scale—anxious arousal subscale (RCMAS: Reynolds & Richmond, 1978); the Screen for Child Anxiety Related Disorders (SCARED: Birmaher et al., 1999); the Spence Child Anxiety Scale (SCAS: Spence, 1998); the State‐Trait Anxiety Inventory for Children (STAI: Spielberger, Gorsuch, Lushene, Vagg, & Jacobs, 1983); and the Social Worries Questionnaire (SWQ: Spence, 1995). A summary of some of the key elements of the included studies is presented in Table 2.

#### 6.1.8 Comparisons to previous reviews

Eight of the studies had not been included in previous reviews of interventions for anxiety in the ASD population, predominantly due to being published after the reviews were completed and because the current review also included theses and dissertations that were experimental or quasi‐experimental studies. Table 3 presents the prior reporting of studies in five previous systematic reviews undertaken by Lang et al. (2010), Sukhodolsky et al. (2013), Kreslins et al. (2015), Ung et al. (2015), and Perihan et al. (2019).

**Table 3:** Comparison of coverage of current review with previous published reviews of literature

	Lang et al. (2010)	Sukhodolsky et al. (2013)	Kreslins et al. (2015)	Ung et al. (2015)	Perihan et al. (2019)
**Chalfant_2007**	x	x	x	x	x
**Clarke_2017**					x
Conaughton_2017					
Corbett_2017					
**Fujii_2013**				x	x
Hepburn_2016					
**Luxford_2017**					x
MacKinnon_2014					
**McConachie_2014**			x	x	x
**McNally Keehn_2013**		x	x	x	x
Murphy_2017					
Ohan_2016					
Piravej_2009					
Pryor_2016					
**Reaven_2009**	x			x	x
**Reaven_2012**		x	x	x	x
**Sofronoff_2005**	x	x	x	x	x
**Storch_2013**		x	x	x	x
**Storch_2015**					x
**Sung_2011**		x	x	x	x
**vanSteensel_2015**					x
**White_2013**		x	x	x	x
**Wood_2009**	x	x	x	x	x
**Wood_2015**				x	x

*Note:* Studies included in other systematic reviews are presented in bold font, while those studies not included in previous reviews are presented in normal font.

Table 4 lists the studies identified in the latest review by Perihan et al. (2019) that were not included in this review with reasons for noninclusion. As can be seen, the main reason for not including studies in this current review is their observational design.

**Table 4:** Studies that were not included in this review but were included in Perihan et al. (2019)

Studies	n	Reasons for exclusion
Drmic, Aljunied, and Reaven (2017)	44	Observational study
Ehrenreich‐May et al. (2014)	20	The BIACA intervention was already covered under the Storch 2015 paper (RCT study) for a larger age group (11 to 16 years); Also, no control group
Maskey, Lowry, Rodgers, McConachie, and Parr (2014)	9	Observational study
Ooi et al. (2008)	6	Observational study (pre‐post design only, no control group)
Scarpa and Reyes (2011)	11	No concurrent diagnosis of anxiety problems and no appropriate measure of anxiety as an outcome
Thomson, Burnham Riosa, and Weiss (2015)	13	Observational (no control group)
Weiss, Viecili, and Bohr (2014)	18	Observational study

#### 6.1.9 Excluded studies

As indicated in Figure 1, 95 of the full‐text papers retrieved were excluded from the current review: 19 did not report levels of anxiety among participants, 16 had no outcome measure of anxiety, 15 did not employ a control or comparison group, 10 had a participant age range out of scope (and no potential for isolating participants within scope), 10 studies were classified as purely observational (pre‐ and postintervention in single group only), seven had no formal diagnosis of ASD in their participants, six were secondary or follow‐up analyses of previous studies that focused on aspect outside of inclusion criteria, four did not include an intervention that met criteria, two were proposals or study protocols only (no results were presented), and another six did not provide sufficient information to satisfy the selection criteria or had other issues. As summarized below and detailed in Appendices B and C in the Supporting Information Material, these 95 excluded articles included those studies that might reasonably have been expected to be included, such as those included in previous reviews, but which did not meet the inclusion criteria of the current review:

- No anxiety present in participants=> 19 studies
- No measurement of anxiety outcomes=> 16 studies
- No control group=> 12 studies
- Age range out of scope=>10 studies
- Observational=> 10 studies
- No ASD diagnosis=> 7 studies
- Secondary study=> 5 studies
- No treatment=> 4 studies
- No comparison group=> 3 studies
- Out of scope=> 3 studies
- Not enough information=> 2 studies
- Protocol/proposal=> 2 studies
- Low quality=> 1 study
- Uses same data as a more recent included study=> 1 study

### 6.2 Risk of bias in included studies

#### 6.2.1 Selection bias

Thirteen of the studies performed adequate random sequence generation, either manually (using staff unrelated to the study, Corbett_2017; Murphy_2017; Sung_2011; White_2013) or generated by computer (Clarke_2017; Luxford_2017; Reaven_2012; Storch_2013; Storch_2015; Wood_2009; Wood_2015). Block randomization procedures, stratified by demographic variables, were used in Fuji_2013, McNally‐Keehn_2013, and Piravej_2009. Despite being identified as randomized controlled trials, Chalfant_2007, Pryor_2016, and Sofronoff_2005 did not provide suf ficient information about randomization methods or procedures to assess potential bias.

It should be noted that four of the included studies were classified as quasi‐experimental studies, in that they included a comparison group but that allocation to groups was not random. Of the quasi‐experimental studies, Hepburn_2016 used a pair‐wise matching scheme for allocation to groups, Ohan_2016 claimed that the order of enrollment “approximated” randomization, while Reaven_2009 had subjects act as their own WLCs.

Allocation concealment was not detailed in the majority of studies, although Conaughton_2017 and Wood_2017 maintaining concealment by conducting baseline measures prior to randomization, and McConachie_2014, Murphy_2017, and Storch_2015 by concealing treatment group allocation from the researchers and independent evaluators throughout the studies.

#### 6.2.2 Performance and detection bias

Performance bias was universally high, due to the nature of the interventions, as it was not possible to blind participants from their treatment group allocation.

Detection bias was higher among studies that used outcome measures based on the reports of participants themselves or their parents (many of whom had also participated in family‐based interventions), but lower in studies that used reports from clinicians or teachers who were blinded to IT allocation. For those studies that used multiple outcome measures from different informants, the risk of performance bias was rated for each outcome measure separately.

#### 6.2.3 Attrition bias

Attrition was rated as low in studies that had little to no attrition in subjects, who evaluated the effect of attrition by comparing therapy completer analyses with “Intent to Treat” analyses (i.e., analyses using original samples and data imputation techniques), or who compared the profile of study dropouts with completers with no statistically significant differences found. Attrition bias was fairly low across the studies, with only Ohan_2016 and Reaven_12 being rated as high. Ohan_2006 reported that six of the treatment families failed to complete their treatment (a loss of 25% of their sample), while Reaven_12 reported a treatment‐completer sample of 47, but only provided baseline data for 43 participants (IT = 20 and TAU = 23) and used “last observation carried forward” imputation for missing data.

#### 6.2.4 Reporting bias

There was no evidence or suggestion of reporting bias in any of the studies. However, a risk of bias assessment was undertaken by rating each study as high, unclear, or low risk of bias against five attributes. A summary of the risk of bias assessment for the included studies is presented alongside each forest‐plot. In addition, publication bias was assessed using a funnel plot.

### 6.3 Synthesis of results

#### 6.3.1 Treatment efficacy

Twenty‐four studies reported outcome measures from one or more informants (clinicians, parents, student self‐reports), with 60 informant reports in total, along with several teacher reports (see Table 2). These studies involved 1,020 participants (524 in the treatment group and 496 in the control group—note that participants in pre‐post studies are counted twice). Rather than averaging all reports within each study across different measures to derive an overall treatment effect, the reports with lower risk of bias were selected. For half of the studies, the clinician report was used. For seven of the studies, the averaged parent‐ and student‐reported SCAS (six instances) or SCARED (one instance) were used. For the other five studies, a parent‐only or student‐only report was used. A random effects meta‐analysis of the 24 studies revealed a statistically significant treatment effect for interventions for reducing anxiety in mainstream school‐aged students with ASD. The overall *SMD* was *d* = −0.83 (95% CI: −1.16, −0.51; *z* = −5.03, *p* < .01) which can be considered a very high effect. Based on these measures, the anxiety levels in the treatment groups were significantly lower than those seen in the control groups at posttreatment.

**Figure 2:**
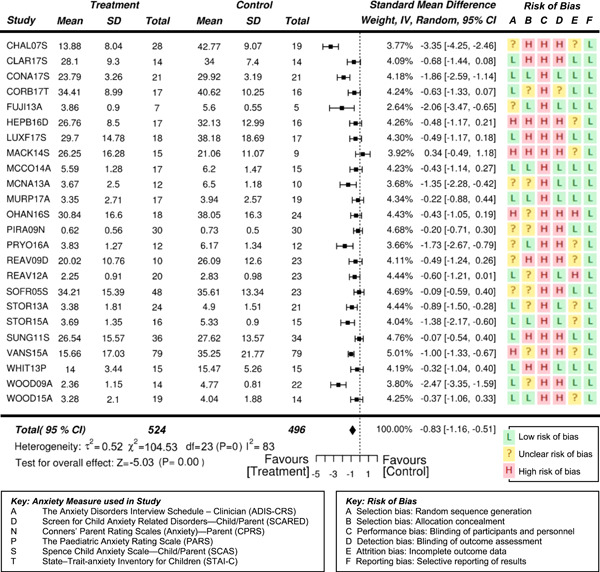
Forest plot of studies included in the meta‐analysis, with risk of bias summary

**Figure 3:**
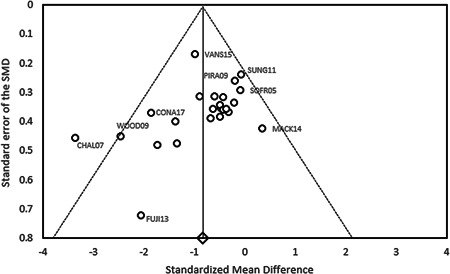
Funnel plot of the 24 included studies

Considerable heterogeneity across the studies and assessments was detected (*I* ^2^ = 83%). Figure 2 presents a forest plot illustrating the results. Visual inspection of the both the forest plot and the funnel plot, as presented in Figure 3, identified the SMD score reported by Chalfant_2007 as an outlier. A sensitivity analysis was carried out by removing this study, reducing the overall *SMD* to −0.71 (95% CI: −0.97, −0.46; *z* = −5.42, *p* < .01). While removal of the outlier did reduce the overall effect size, the difference between treatment and control conditions at posttreatment remained very high and statistically significant.

#### 6.3.2 Clinician reported outcome measures

**Figure 4:**
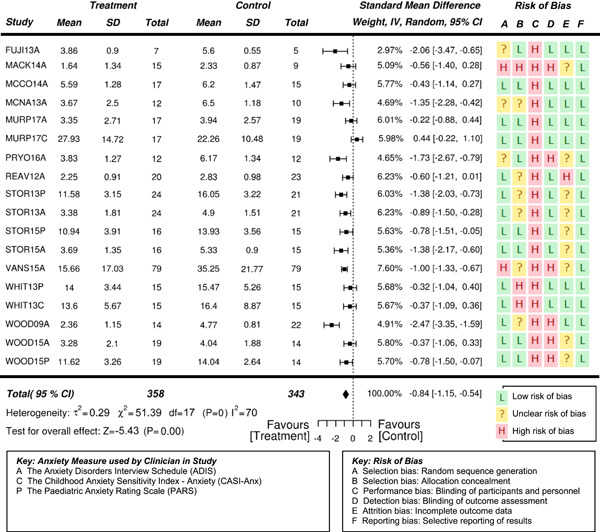
Forest plot of clinician‐reported outcome measures with risk of bias summary

Thirteen studies involving a total of 526 students (267 in the treatment condition and 259 in the control condition) reported one or more clinician‐reported outcome measure, with 18 reports in total. The three measures used by clinicians were the ADIS (A, 12 instances), the CASI‐Anx (C, 2 instances), and the PARS (P, 4 instances). All studies reported greater improvements posttreatment in the treatment condition compared to the control condition, although Murphy_2017) reported nonsignificant mixed results. The overall *SMD* was *d* = −0.84 (95% CI: −1.15, −0.54; *z* = −5.43, *p* < .01) which can be considered a very high effect, potentially equivalent to 10 months developmental progress. Based on these measures, the anxiety levels in the treatment groups were significantly lower than those seen in the control groups at posttreatment. Considerable heterogeneity across the studies and assessments was detected (*I* ^2^ = 70%). Figure 4 presents a forest plot illustrating the results.

#### 6.3.3 Parent reported outcome measures

**Figure 5:**
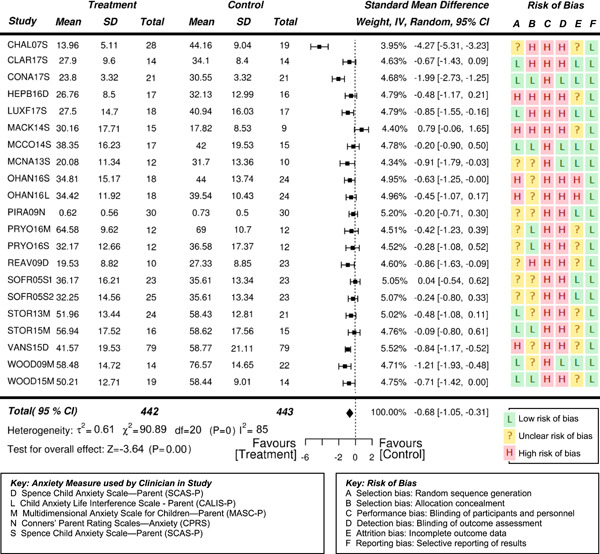
Forest plot of parent‐reported outcome measures with risk of bias summary

Nineteen studies reported one or more parent reported outcome measures, with 21 reports in total. These studies involved 819 participants (412 in the treatment group and 407 in the control group). The five measures used with parents were the SCAS (S, 11 instances), the MASC (M, 5 instances), SCARED (D, 3 instances), CALIS (C, 1 instance), and the CPRS (N, 1 instance). The overall *SMD* was *d* = −0.68 (95% CI: −1.05, −0.31); *z* = −3.64, *p* < .01), indicating that the difference between the treatment and control groups at posttreatment reached significance. There was significant heterogeneity across the included studies and measures (*I* ^2^ = 85%). Figure 5 presents a forest plot of the results, along with a summary of the risk of bias. Like other systematic reviews (e.g., Kreslins et al., 2015), the SMD reported by Chalfant_2007 was assessed as an outlier, being substantially higher than the others reported. A sensitivity analysis was carried out. Once this outlier was removed, the overall *SMD* decreased to −0.53 (95%CI −0.76, −0.31; *z* = −4.73, *p* < .01). Although the summary estimate decreased following removal of the outlying study, the high treatment effect remained statistically significant and potentially equivalent to 7‐month developmental progress.

#### 6.3.4 Student self‐reported outcome measures

**Figure 6:**
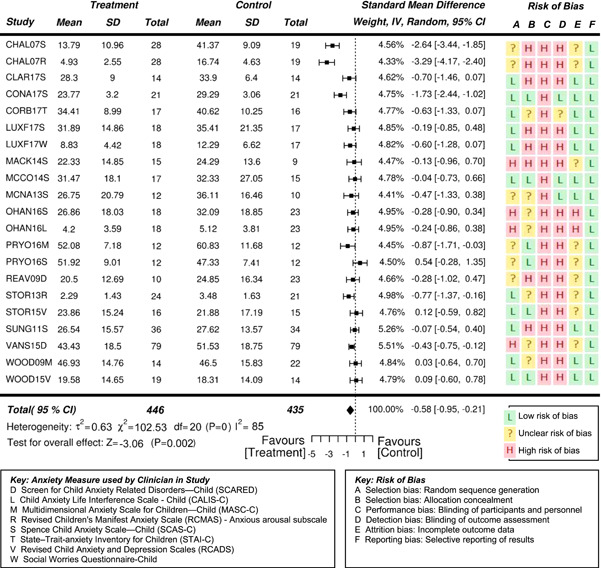
Forest plot of self‐reported outcome measures with risk of bias summary

Self‐reported outcome data from 734 student participants (370 in the treatment group and 364 in the control group) from 21 outcome reports across 17 studies were synthesized. The main student outcome measure used by 10 studies was the SCAS (S). The SCARED (D), MASC (M), RCMAS (R), and RCADS (V) had each been used by two of the studies, while the CALIS (L), STAI (T), and SWQ (W) had each been used by one of the studies. The overall *SMD* was *d* = −0.58 (95% CI: −0.95, −0.21; *z* = −3.06, *p* = .002) with a significant difference (high effect size, equivalent to 7‐month progress) between the treatment and control conditions at posttreatment. Figure 6 presents a forest plot of the student results. There were high levels of heterogeneity across the studies (*I* ^2^ = 85%). The two *SMD* scores reported by Chalfant_2007 were again identified as outliers and a sensitivity analysis was undertaken. Removal of this study reduced the overall *SMD* to −0.35 (95% CI: −0.55, − 0.15; *z* = −3.41, *p* < .01). This difference between treatment and control conditions at posttreatment of a moderate effect was significant and equivalent to 4‐month progress.

#### 6.3.5 Moderator analysis

Also of interest in this study, was the potential moderating effects of a) family involvement in sessions and b) the individual or group nature of sessions. These two moderators were analyzed to help address the lack of similar investigations in previous reviews published, and to draw comparison to the most recent review by Perihan et al. (2019), which did consider the potential moderator of parental involvement.

#### 6.3.6 Family involvement

**Figure 7:**
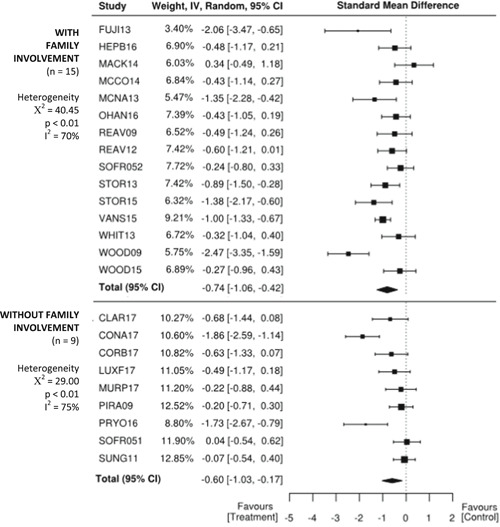
Forest plot comparing treatments with and without family involvement

Two groups were created based on family involvement, in order to compare the outcomes of treatments with parental involvement (*n* = 15, excluding outlier Chalfant_2007) to treatments without parental involvement (*n* = 9). It should be noted that the Piravej et al. (2009) study involving Thai massage was categorized as being without family involvement even though for safety reasons, the parent was present in the room. Similar to Perihan et al.'s (2019) findings, treatments that had family involvement resulted in a larger overall effect size than treatments without parental involvement, a difference equivalent to 2‐month additional progress. The *SMD* with family involvement yielded a very high significant effect size of *d* = −0.74 (95% CI: −1.06, −0.42; *z* = −4.55, *p* < .01). In comparison, the *SMD* without family involvement resulted in a high significant effect size of *d* = −0.60 (95% CI: −1.03, −0.17; *z* = −2.73, *p* = .006). There were high levels of heterogeneity across the two groups of studies (*I* ^2^ = 70% with involvement; 75% without involvement). Figure 7 presents a forest plot of the moderating effect of family involvement.

#### 6.3.7 Individual or group treatment

**Figure 8:**
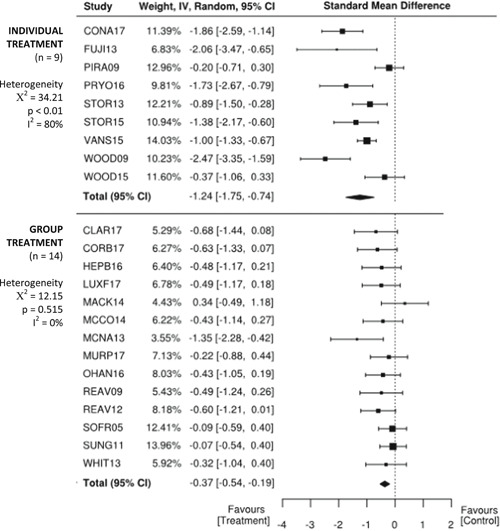
Forest plot comparing treatments that involve individual or group participation

In order to compare the potential moderating effect of treatment format, two groups of studies were formed and assessed based on whether the treatment was administered on an individual basis (*n* = 9), like the Thai massage (Piravej et al., 2009), or in a group setting (*n* = 14), such as the peer‐mediated, theater‐based intervention (Corbett et al., 2017). The forest plot presented in Figure 8 suggests that individually based treatments resulted in a larger overall effect size than group‐based treatments, a difference equivalent to 7‐month additional progress. The *SMD* yielded a very high significant effect size of *d* = −1.24 (95% CI: −1.75, −0.74; *z* = −4.87, *p* < .01). In comparison, the *SMD* for group treatments resulted in a moderate significant effect size of *d* = −0.37 (95% CI: −0.54, −0.19; z = −4.10, *p* < .01). There was a high level of heterogeneity across studies involving individual treatments (*I* ^2^ = 80%) and a low level of heterogeneity across the studies involving group treatments (*I* ^2^ = 0%).

## 7 DISCUSSION

### 7.1 Summary of main results

Eighteen RCTs and six quasi‐experimental studies evaluating the effects of interventions targeting anxiety for individuals of mainstream school age—with participants in some study also involving slightly younger and slightly older young people—with ASD were included in this review. The results of the meta‐analyses provide some evidence that psychoeducational interventions for anxiety, predominantly CBT, may improve anxiety symptoms (*SMD* = −0.71, *p* < .01, after removal of outliers) for mainstream school‐aged children with ASD, ultimately reducing the number of diagnoses of anxiety disorders for some participants. More favorable outcomes for the treatment group compared to the control groups were recorded across the majority of studies, with an overall moderate effect size. Outcomes varied by respondent group, however, with larger effect sizes recorded when outcome measures were based on clinician reports (*SMD* = −0.84, *p* < .01), compared to parent reports (*SMD* = −0.53, *p* < .01). Difference between treatment and control groups were smaller again, although still significant, when the participants reported outcomes themselves (*SMD* = −0.35, *p* = .001). These differences between respondents have been noted elsewhere, and may introduce bias to the results (underestimating the effects). Moderators indicated larger effects for treatments that involved parents (*SMD* = −0.74, *p* < .01) than for student‐only interventions (*SMD* = −0.60, *p* < .01). Treatments that were administered individually one‐on‐one (*SMD* = −1.24, *p* < .01), indicated larger effects than for treatments delivered at a group‐level (*SMD* = −0.37, *p* < .01).

### 7.2 Overall completeness and applicability of evidence

The large number of studies meeting our original inclusion criteria was of sufficient size to warrant restricting the results to a meta‐analysis of RCTs and quasi‐experimental studies. Data on the primary outcome—anxiety—was gathered using a variety of instruments. Although this may be seen as a source of variability and imprecision in the results, all of the instruments used adhered to published recommendations for reliability and quality (Lecavalier et al., 2014; Wigham & McConachie, 2014) and have been analyzed in previous reviews (Kreslins et al., 2015).

The majority of included studies were located in developed, Western countries, and the interventions conducted in clinical settings attached to medical or university institutions. Only two studies examined home‐based interventions and two interventions were conducted in schools. This is probably an accurate reflection of the state of research of this nature, but did impact on our ability to examine intervention settings as a source of variability in effectiveness. Almost twice as many studies involved treatments with parental involvement and there tended to more studies that involved treatments delivered in a group format.

Similarly, while a small number of studies included outcome measures based on the reports of teachers, these were usually limited by significant attrition and thus recommended extreme caution in interpreting results, or did not report results for this respondent group. Given our proposed focus on real‐world settings, the inability to evaluate the effectiveness of interventions based on the view of teachers is an unfortunate limitation to this review.

Only two of the included studies used an intervention other than CBT. While CBT is a more widely accepted intervention for anxiety, including for participants with ASD, it was not our intention to limit the studies to CBT. It is possible that the focus on RCTs and quasi‐experimental studies unintentionally limited coverage of studies using more recently developed interventions, which may still be at the stage of case studies or observational studies.

Finally, while it was not a requirement for inclusion in the current review, the majority of the studies limited participants to individuals with functioning above a certain level of cognitive ability, most commonly a full scale or verbal IQ of 70 or above, or in one case, the ability to read and write (in English) at an 8‐year‐old level as a minimum. These restrictions were explained as being due to the demands of CBT. Only three studies did not place restrictions (either explicitly or de facto by requiring that participants be attending a mainstream school) on the cognitive functioning of participants, and two of these employed cognitive‐behavioral therapy‐based interventions (Hepburn_2016; MacKinnon_2014). Neither study examined the potential impact of cognitive functioning on anxiety outcomes and did not report outcomes for participants grouped by cognitive functioning level. This does limit our ability to determine what effects these types of interventions might have on individuals with ASD and intellectual disability. This limitation has been noted in other research focusing on the effectiveness of psychosocial interventions in populations with ASD (Kreslin et al., 2015).

### 7.3 Quality of the evidence

The quality of the evidence can be considered moderate. Results in favor of treatment groups compared to control groups were fairly consistent across the included studies, although a number noted mixed nonsignificant results when using reports from more than one respondent (particularly, when using parent and self‐reports).

Overall, the number of low and questionable assessments of risk outweighed those of high risk. There are, however, issues around the variety of outcome measures and informants that may introduce imprecision to the overall measures of effectiveness and thus should be considered.

The effectiveness of the interventions was generally stronger for clinician (mostly blinded) reports but lower among parent and self‐report measures. Issues with self‐report in ASD children have been noted in other reviews, with both Kreslins et al. (2015) and Sukhodolsky et al. (2013) reporting lower effect sizes when using self‐report as the outcome measure of anxiety. It is difficult to identify whether these differences emerge from difficulties in interpreting the questions on the outcome measures or may reflect a lower level of insight into their own symptoms in this particular population. It is worth noting that none of the self‐report outcomes measures had been designed, modified or normed for use with children with ASD.

Due to the nature of the interventions and the selected outcome measures, the risk of performance and detection bias were higher for some outcome measures (parent and self‐reports) compared to others. Apart from Clarke_2017, which was a school‐based intervention and included a parent report as an outcome measure, parents were aware of the treatment group their child was assigned to. Accordingly, the nature of the interventions made it impossible to blind participants themselves to treatment status, so all self‐reports may reflect high performance and detection bias. Given this high risk of bias, the results, particularly those based on parent or self‐report should be interpreted with caution.

### 7.4 Limitations and potential biases in the review process

Although the systematic nature of the review process followed here, decreases the potential for bias, risks of bias in the review process remain. The greatest risk of bias of this review was the selection of studies, specifically, the decision to limit the inclusion criteria to randomized control studies and quasi‐experimental studies. The inclusion of RCTs alongside quasi‐experimental studies, and studies of CBT alongside other interventions, may have introduced variability to the estimates of effectiveness and is thus a potential limitation to the results.

### 7.5 Agreements and disagreements with other studies or reviews

The results of this review and meta‐analysis suggest there is moderately strong evidence for the effectiveness of psychosocial interventions, such as CBT, in reducing anxiety amongst mainstream school‐aged children with ASD. This is consistent with the conclusions of similar reviews (Kreslins et al., 2015; Sukhodolsky et al., 2013; Ung et al., 2015), although these reviews focused solely on CBT, while this review included other approaches.

The results of the current review do differ from previous reviews, however, in finding that the interventions were effective even from the point of view of the children participating in them. This meta‐analysis found an *SMD* of −0.35, *p* = .001, when using self‐report as the outcome measure, compared to *d* = −0.65 (*p* = .10) in Kreslins et al. (2015) and *d* = −0.68 (*p *= .12) in Sukhodolsky et al. (2013). The moderator of family involvement (*SMD*_with_ = −0.74, *p* < .01; *SMD*_without_ = −0.60, *p* < .01) was in keeping with Perihan et al.'s (2019) findings (*g*_with_ = −0.85, *p* < .05; *g*_without_ = −0.34, *p* < .05).

The results of this review are, thus, in line with previous reviews, and suggest that interventions can have positive effects on the experiences of young people with ASD and anxiety, although more research is needed to identify the underlying cause or causes of the discrepancy in effectiveness depending on the respondent.

## 8 AUTHORS’ CONCLUSIONS

### 8.1 Implications for practice and policy

CBT in various forms is one of the most widely used psychoeducational interventions to improve the anxiety symptoms of mainstream school‐aged children with ASD. The results of the meta‐analyses in this review suggest that children who participate in interventions based on CBT, whether modified specifically for those with ASD or not, may make significant gains in terms of reduction of anxiety symptoms, in some cases no longer meeting criteria for a primary anxiety diagnosis or comorbid diagnoses of other anxiety disorders. Evidence in support of other psychoeducational interventions, such as massage and theater therapy to address social anxiety, is more limited, not just due to the popularity of CBT but also due to the quality of the smaller number of non‐CBT studies available.

While the review does indicate that interventions based on the principles of CBT may be effective for reducing anxiety, the variety of curricula (and modifications made to those curricula) used in the studies included may have confounded the results. There were not a sufficient number of studies that employed the same curricula (even those that used the same base curricula may have made different modifications) to allow for a direct comparison of effects between curricula, and so it is not possible to provide recommendations as to whether one CBT curricula might be more effective than another, or indeed whether any one program may be more effective with a certain subgroup of participants (e.g., individuals with an intellectual disability, ADHD). However, as CBT provides an overarching theoretical framework upon which curricula are based, different programs that are grounded in the principles of CBT should, at least in principle, have similar degrees of effectiveness.

### 8.2 Implications for research

The results of this review suggest that while there is evidence that CBT is an effective behavioral treatment for anxiety in some children and youth with ASD, there is still work to be done in terms of identifying the characteristics of these interventions that contribute to their effectiveness and identifying the characteristics of participants who are more likely to respond to such interventions. The results suggest that group therapies are less effective than individual one‐on‐one therapies and that having the family involved is more effect at reducing anxiety than not. Many of the studies reviewed here included modifications to published curricula that were hypothesized to improve the acceptance and effectiveness of the interventions for children with ASD, such as visual aids, highly structured sessions and flexibility around the number of length of sessions. It is unclear what effect, if any, the use of different curricula and modifications had on the results of this review. Future research with larger samples and active control groups are necessary to allow direct comparisons of the different curricula and identification of the characteristics of participants (e.g., age, social competence, level of language ability, and communication skills) for whom the intervention is most likely to be of benefit.

Research should also focus on expanding the cognitive functioning levels of participants (i.e., including individuals with below average cognitive abilities) so as to identify the characteristics of interventions that can be employed with children and adolescents with ASD and lower levels of general functioning or language ability.

The trend of somewhat weaker effects of interventions when outcomes are measured via self‐report, noted here and in similar reviews (e.g., Kreslins et al., 2015; Sukhodolsky et al., 2013) warrants further investigation. It may be that this trend reflects actual differences in the perceived benefits of interventions between clinicians, parents and children. However, it is troubling to assume that the more valid assessment of a child's experience is an external observer, whether that be a clinician or a parent. Other factors, such as the development of self‐awareness and understanding of health concepts, have been suggested by other researchers as potential sources of inconsistency in self‐reports with children, and more particularly with children with ASD (Kreslins et al., 2015).

Some suggest that individuals with ASD can manifest in ways that are idiosyncratic and not aligned with the ways that anxiety presents in non‐ASD populations (Kerns & Kendall, 2012; Kerns et al., 2016; Uljarević et al., 2018). Certain traits and behaviors, such as social avoidance or rigid, ritualistic behaviors, can be a manifestation of both core ASD symptomatology and indicative of comorbid anxiety (Kerns et al., 2015). However, with rare exception (see Rodgers et al., 2016), instruments used to measure anxiety in ASD have been develop for non‐ASD populations. Such instruments are not designed to be sensitive to distinguishing atypical anxiety presentations in ASD populations nor distinguish whether a particular symptom is a presentation of ASD or comorbid anxiety—nor should they be. Nevertheless, current instruments might at the same time both over‐ and undersample anxiety problems associated with ASD. What may be of importance, however, is whether symptoms are more or less responsive to intervention among populations with anxiety alone versus those with ASD and comorbid anxiety, which might be more ingrained. Accordingly, the development of instruments for anxiety symptoms that are specifically designed for and normed with ASD population may be of use.

It may be worth considering the input of those with ASD in the design and modifications of interventions for anxiety. As with other groups, it is important to involve people with ASD in the design and modification of interventions designed for them, including those targeting anxiety. People with ASD are likely to be able to shed important insight into the strengths and weaknesses of different approaches, and also to the specific skills required of therapists working with this population. Finally, it is imperative to develop instruments that are valid in assessing treatment effects in people with ASD. Assessing participants’ satisfaction with interventions is not enough, as they are unlikely to have been exposed to multiple interventions at the same time and thus be able to make direct comparisons. Instead, children and adolescents with ASD should be able to provide formative feedback and be involved in redesign.

## CONFLICT OF INTERESTS

The authors declare that there are no conflict of interests.

## AUTHOR CONTRIBUTIONS

K. H., K. D., K. A., P. L., E. O'Gr., M. U., G. V., and D. H. contributed to the writing and revising of the original study protocol as well as this report. K. D. and K. A. are accredited JBI systematic reviewers. The search strategy was developed with Trevitt, Senior Librarian of the Cunningham Library at the Australian Council for Educational Research (ACER). P. L. was responsible for finalizing this review in response to reviewers’ comments.

## Supporting information

Supporting information

## Footnotes

^1^
Studies included in this review are cited by using first‐author_date, rather than APA style.
