# Supplementary material for: Interventions for anxiety in mainstream school‐aged children with autism spectrum disorder: A systematic review
Source: Campbell Syst Rev. 2020 May 5;16(2):e1086. doi: 10.1002/cl2.1086 (PMC8356281; doi:10.1002/cl2.1086)

# Online supplements

## Appendix A: Customized search statements for bibliographic databases

| **Academic Research Complete (EBSCO)** | |
| --- | --- |
| Date of search: 25 May 2017  Search update: 31December 2017 | |
| **Search**  **Sets** | **Search Queries**  Search Mode: Boolean/Phrase. Related Terms Not Applied.  Date Range: 1996-2017 |
| S7 | S1 AND S2 AND S5 AND S6 |
| S6 | Intervention OR treatment OR therap* OR psychotherap* |
| S5 | S3 OR S4 |
| S4 | SU ( children OR adolescence OR youth OR teenagers OR boys OR girls ) OR ( young people OR young person ) |
| S3 | SU students AND SU school* |
| S2 | SU ( anxiety OR fear ) OR ( anxious OR internal* ) |
| S1 | SU ( AUTIS* OR ASPERGER'S syndrome ) OR ( ASD OR Pervasive Developmental Disorder OR PDD NOS OR PDD unspecified ) |

| **A+Education (Informit)** | |
| --- | --- |
| Date of search: 7 June 2017  Search update: 31 December 2017 | |
| **Search Sets** | **Search Queries**  Date Range: 1996-2017 |
| s3 | #1 AND #2 |
| S2 | Anxiety OR anxious OR internali* OR fear  Includes Subject Terms:   - Anxiety - Fear |
| S1 | ASD OR Asperger* OR autis* OR "Pervasive Developmental" OR "PDD NOS" OR "PDD unspecified"  Includes Subject Terms:   - Autism spectrum disorders - Asperger syndrome |

| **British Education Index (EBSCO)** | |
| --- | --- |
| Date of search: 25 May 2017  Updates via ongoing alerts | |
| **Search Sets** | **Search Queries**  Search Mode: Boolean/Phrase. Related Terms Not Applied.  Date Range: 1996-2017 |
| S7 | S15 AND S16 AND S19 AND S20 |
| S6 | Intervention OR treatment OR therap* OR psychotherap* OR evaluation OR outcome OR program OR trial* OR experimental OR control group OR random* OR best practi* or evidence |
| S5 | S3 OR S4 |
| S4 | SU students AND SU school* |
| S3 | child* OR adolescen* OR preadolescen* OR pre adolescen* OR youth OR teen* OR teen age* OR young people OR young person OR boy OR girl |
| S2 | Anxiety OR anxious OR internali* OR fear  Includes Subject Terms:   - Anxiety - Fear |
| S1 | ASD OR Asperger* OR autis* OR Pervasive Developmental Disorder OR PDD NOS OR PDD unspecified  Includes Subject Terms:   - AUTISM - AUTISM spectrum disorders - ASPERGER'S syndrome - AUTISM in children - AUTISTIC children - Autism in adolescence - PERVASIVE developmental disorder not otherwise specified |

| **CBCA Complete (Proquest)** | |
| --- | --- |
| Date of search: 1 June 2017  Search update: 31 December 2017 | |
| **Search Sets** | **Search Queries**  Date Range: 1996-2017 |
| S5 | S1 AND S2 AND S3 AND S4 |
| S4 | all(Intervention) OR all(treatment) OR all(therap*) OR all(psychotherap*) OR all(evaluation) OR all(outcome) OR all(program) OR all(trial*) OR all(experimental) OR all(control group) OR all(random*) OR all(best practi*) or all(evidence based) |
| S3 | all(Student OR child* OR adolescen* OR preadolescen* OR pre-adolescen* OR youth OR teen* OR teen age* OR young people OR young person OR boy OR girl ) |
| S2 | (all(Anxiet*) OR all(anxious) OR all(internali*) OR all(fear))  Includes Subject Terms:   - Anxieties - Fear & Phobias |
| S1 | all(ASD OR Asperger* OR autis* OR Pervasive Developmental Disorder OR PDD NOS OR PDD unspecified)  Includes Subject Terms:   - Autism - Asperger syndrome |

| **CINAHL Plus with Full Text (EBSCO)** | |
| --- | --- |
| Date of search: 5 June 2017  Search update: 31 December 2017 | |
| **Search Sets** | **Search Queries**  Search Mode: Boolean/Phrase. Related Terms Not Applied.  Date Range: 1996-2017 |
| S5 | S1 AND S2 AND S3 AND S4 |
| S4 | Intervention OR treatment OR therap* OR psychotherap* OR evaluation OR outcome OR program OR trial* OR experimental OR control group OR random* OR best practi* or evidence based |
| S3 | Student OR child* OR adolescen* OR preadolescen* OR pre adolescen* OR youth OR teen* OR teen age* OR young people OR young person OR boy OR girl  AND  Age Groups: Child, Preschool: 2-5 years, Child: 6-12 years, Adolescent: 13-18 years, All Child |
| S2 | SU ( Anxiety OR Fear OR Anxiety Disorders) OR ( Anxious* OR internali* ) |
| S1 | SU autistic disorder OR ( ASD OR Asperger* OR autis* OR Pervasive Developmental Disorder OR PDD NOS OR PDD unspecified )  Includes Subject Term:   - Pervasive Developmental Disorder-Not Otherwise Specified |

| **Education Research Complete (EBSCO)** | |
| --- | --- |
| Date of search: 26 May 2017  Updates via ongoing alerts | |
| **Search Sets** | **Search Queries**  Search Mode: Boolean/Phrase. Related Terms Not Applied.  Date Range: 1996-2017 |
| S5 | S1 AND S2 AND S3 AND S4 |
| S4 | Intervention OR treatment OR therap* OR psychotherap* OR evaluation OR outcome OR program OR trial* OR experimental OR control group OR random* OR best practi* or evidence based |
| S3 | (SU students AND SU school*) OR (child* OR adolescen* OR preadolescen* OR pre adolescen* OR youth OR teen* OR teen age* OR preteen OR young people OR young person OR boy OR girl) |
| S2 | SU (Anxiety OR Fear ) OR (anxious OR internali*) |
| S1 | SU ( Autis* OR ASPERGER'S syndrome ) OR ( ASD OR Pervasive Developmental Disorder OR PDD NOS OR PDD unspecified ) |

| **EMBASE (Elsevier)** | |
| --- | --- |
| Date of search: 7 June 2017  Search update: 31 December 2017 | |
| **Search Sets** | **Search Queries**  Date Range: 1996-2017 |
| S7 | S6 AND ([adolescent]/lim OR [child]/lim OR [preschool]/lim OR [school]/lim OR [young adult]/lim) |
| S6 | S1 AND S4 AND S5 |
| S5 | 'intervention'/exp OR 'treatment'/exp OR 'therapy'/exp OR 'psychotherapy'/ |
| S4 | S2 OR S3 |
| S3 | internalis* OR internaliz* |
| S2 | 'anxiety'/de OR 'anxiety disorder'/de OR 'fear'/ |
| S1 | 'autism'/de OR 'asperger syndrome'/de OR 'pervasive developmental disorder not otherwise specified'/de |

| **ERIC (EBSCO)** | |
| --- | --- |
| Date of search: 25 May 2017  Updates via ongoing alerts | |
| **Search**  **Sets** | **Search Queries**  Search Mode: Boolean/Phrase. Related Terms Not Applied.  Date Range: 1996-2017 |
| S7 | S1 AND S2 AND S3 AND S6 |
| S6 | S4 OR S5 |
| S5 | (Student OR child* OR adolescen* OR preadolescen* OR pre adolescen* OR youth OR teen* OR teen age* OR young people OR young person OR boy OR girl) AND  Educational Level: Early Childhood Education, Elementary Education, Elementary Secondary Education, Grade 1, Grade 2, Grade 3, Grade 4, Grade 5, Grade 6, Grade 7, Grade 8, Grade 9, Grade 10, Grade 11, Grade 12, High School Equivalency Programs, High Schools, Intermediate Grades, Junior High Schools, Kindergarten, Middle Schools, Preschool Education, Primary Education, Secondary Education |
| S4 | DE (Children OR Young Children OR Adolescents OR Preadolescents OR Youth OR High School Students OR Elementary School Students OR Middle School Students OR Secondary School Students OR Junior High School Students) |
| S3 | (Intervention OR treatment OR therap* OR psychotherap* OR evaluation OR outcome OR program* OR trial* OR experimental OR quasiexperimental OR control group OR random* OR Best practi* or evidence based) |
| S2 | (Anxiety OR anxious OR internali* OR fear )  Includes Subject Terms:   - Anxiety - Fear - Anxiety Disorders |
| S1 | (ASD OR Asperger* OR autis* OR Pervasive Developmental Disorder OR PDD NOS OR PDD unspecified)  Includes Subject Terms:   - Autism - Pervasive Developmental Disorders - Asperger Syndrome |

| **PsycInfo (EBSCO)** | |
| --- | --- |
| Date of search: 4 May 2017  Updates via ongoing alerts | |
| **Search**  **Sets** | **Search Queries**  Search Mode: Boolean/Phrase. Related Terms Not Applied.  Date Range: 1996-2017 |
| S7 | S1 AND S2 AND S5 AND S6 |
| S6 | S3 OR S4 |
| S5 | SU ( Intervention OR treatment OR therap* OR psychotherap* OR evaluation OR outcomes OR program OR experimental OR "experiment controls" OR "random sampling" OR "best practices" or "evidence based practices" ) OR TI ( Intervention OR treatment OR therap* OR psychotherap* ) OR AB ( Intervention OR treatment OR therap* OR psychotherap* ) |
| S4 | (student OR child* OR adolescen* OR preadolescen* OR pre adolescen* OR youth OR teen* OR teen age* OR young people OR young person OR boy OR girl)  AND  Age Groups:   - Childhood (birth-12 yrs), - Preschool Age (2-5 yrs), S - chool Age (6-12 yrs), - Adolescence (13-17 yrs), - Young Adulthood (18-29 yrs) |
| S3 | DE Kindergarten Students OR Preschool Students OR Elementary School Students OR Intermediate School Students OR Primary School Students OR Middle School Students OR High School Students OR Junior High School Students |
| S2 | SU ( Anxiety OR Fear OR Internalization ) OR TI ( anxious OR internali*) OR AB ( anxious OR internali* ) |
| S1 | DE Autism Spectrum Disorders OR TI ( ASD OR Asperger* OR autis* OR Pervasive Developmental Disorder OR PDD NOS OR PDD unspecified ) OR AB ( ASD OR Asperger* OR autis* OR Pervasive Developmental Disorder OR PDD NOS OR PDD unspecified ) |

| **PubMed** | |
| --- | --- |
| Date of search: 5 June 2017  Updates via ongoing alerts | |
| **Search Sets** | **Search Queries**  Date Range: 1996-2017 |
| S10 | (S9 AND S8 AND S4AND S3) |
| S9 | (therapeutics[Text Word] OR intervention[Text Word] OR therapy[Text Word] OR psychotherapy[Text Word] OR treatment[Text Word]) |
| S8 | (S5 OR S6 OR S7) |
| S7 | (child*[Title/Abstract] OR adolescen*[Title/Abstract] OR preadolescen*[Title/Abstract] OR pre adolescen*[Title/Abstract] OR youth[Title/Abstract] OR teen*[Title/Abstract] OR teen age*[Title/Abstract] OR young people[Title/Abstract] OR young person[Title/Abstract] OR girl*[Title/Abstract] OR boy*[Title/Abstract]) |
| S6 | (STUDENT*[Title/Abstract]) AND SCHOOL*[Title/Abstract] |
| S5 | ("adolescent"[MeSH Terms]) OR "child"[MeSH Terms] |
| S4 | ("anxiety"[MeSH Terms] OR "anxiety disorders"[MeSH Terms] OR "fear"[MeSH Terms]) OR (internaliz*[Title/Abstract] OR internalis*[Title/Abstract] OR anxious[Title/Abstract] OR anxiety[Title/Abstract] OR fear[Title/Abstract]) |
| S3 | (S1 OR S2) |
| S2 | (ASD[Title/Abstract] OR autism[Title/Abstract] OR autistic[Title/Abstract] OR Asperger*[Title/Abstract] OR "PDD NOS"[Title/Abstract] OR "PDD unspecified"[Title/Abstract]) |
| S1 | "child development disorders, pervasive"[MeSH Terms] |

| **SCOPUS (Elsevier)** | |
| --- | --- |
| Date of search: 11 May 2017  Updates via ongoing alerts | |
| **Search**  **Set** | **Search Queries**  No Thesaurus available. Date Range: 1996-2017 |
| S5 | S1 AND S2 AND S3 AND S4  AND  Collection Limiters:   - Social Sciences - Arts & Humanities - Neuroscience |
| S4 | TITLE-ABS-KEY  (intervention OR treatment OR therap* OR psychotherap* OR evaluation OR outcome OR program* OR trial* OR experimental OR (control W/0 group) OR random* OR (best W/0 practi*) OR "evidence based") |
| S3 | TITLE-ABS-KEY  (student OR child* OR adolescen* OR preadolescen* OR (pre W/0 adolescen*) OR youth OR teen* OR (teen W/0 age*) OR "young people" OR "young person" OR boy OR girl) |
| S2 | TITLE-ABS-KEY  (anxiety OR anxious OR internali* OR fear) |
| S1 | TITLE-ABS-KEY  (asd OR asperger* OR autis* OR "Pervasive Developmental" W/0 disorder* OR "PDD NOS" OR "PDD unspecified) |

| **SocIndex (EBSCO)** | |
| --- | --- |
| Date of search: 26 May 2017  Updates via ongoing alerts | |
| **Search Sets** | **Search Queries**  Search Mode: Boolean/Phrase. Related Terms Not Applied.  Date Range: 1996-2017 |
| S5 | (S1 AND S2 AND S3 AND S4) |
| S4 | Intervention OR treatment OR therap* OR psychotherap* OR evaluation OR outcome OR program OR trial* OR experimental OR control group OR random* OR best practi* or evidence based |
| S3 | Student OR child* OR adolescen* OR preadolescen* OR pre adolescen* OR youth OR teen* OR teen age* OR young people OR young person OR boy OR girl |
| S2 | Anxiety OR anxious OR internali* OR fear |
| S1 | ASD OR Asperger* OR autis* OR Pervasive Developmental Disorder OR PDD NOS OR PDD unspecified |

## Appendix B: Critical appraisal checklist for experimental (RCT) studies and results of excluded studies


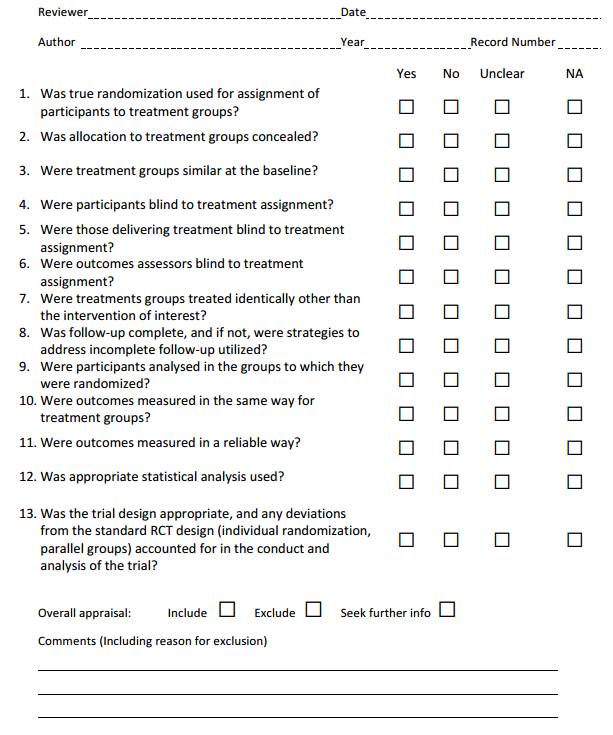


Source:
<http://joannabriggs.org/assets/docs/critical-appraisal-tools/JBI_Critical_Appraisal-Checklist_for_Randomized_Controlled_Trials.pdf>

| **Excluded Experimental (RCT) study** | **Q1** | **Q2** | **Q3** | **Q4** | **Q5** | **Q6** | **Q7** | **Q8** | **Q9** | **Q10** | **Q11** | **Q12** | **Q13** | **Notes** |
| --- | --- | --- | --- | --- | --- | --- | --- | --- | --- | --- | --- | --- | --- | --- |
| Aman, MG, Mcdougle, CJ, Scahill, L, Handen, B, Arnold, LE, Johnson, C, & Sukhodolsky, DD 2009 | Y | Y | Y | N | N | Y | Y | Y | Y | Y | Y | Y | Y | NA |
| Aronoff, E, Hillyer, R, & Leon, M 2016 | N | NA | NA | NA | NA | NA | NA | NA | NA | NA | Y | Y | NA | Not RCT, poor quality |
| Boyer, BE, Doove, LL, Geurts, HM, Prins, PJ, Van Mechelen, I, & Van der Oord, S 2016 | Y | Y | Y | N | N | N | Y | Y | Y | Y | Y | Y | Y | NA |
| Davison, K, Bowling, A, Garcia, J, Wood, B, Hermesch, R, Prince, J, & Slavet, J 2016 | Y | N | U | N | N | N | U | NA | NA | NA | NA | NA | NA | Anxiety not an outcome |
| Drahota, A, Wood, JJ, Sze, KM, & Van Dyke, M 2011 | Y | Y | Y | Y | Y | Y | Y | Y | Y | NA | NA | NA | NA | Anxiety is not an outcome - its comorbid |
| Edelson, SM, Edelson, MG, Kerr, DC, & Grandin, T 1999 | Y | U | Y | Y | Y | N | NA | NA | NA | N | NA | NA | NA | poor quality |
| Elliott, N, Koegel, L, Gore, M, & McCleery, JP 2017 | Y | U | U | N | N | Y | Y | Y | Y | Y | Y | U | Y | this is a poster so info is limited |
| Freitag, CM, Cholemkery, H, Elsuni, L, Kroeger, AK, Bender, S, Kunz, CU, & Kieser, M 2013 | NA | N | NA | NA | NA | NA | NA | NA | NA | NA | NA | NA | NA | This is a protocol for RCT - not the RCT itself |
| Halliwell, N 2017 | NA | N | NA | NA | NA | NA | NA | NA | NA | NA | NA | NA | NA | This is an appraisal of Luxford's paper |
| Johnson, N, Bree, O, Lalley, EE, Rettler, K, Grande, P, Gani, MO, & Ahamed, SI 2014 | Y | Y | U | N | N | Y | Y | Y | Y | Y | Y | Y | Y | NA |
| Kelly, RM 2015 | Y | N | NA | NA | NA | NA | NA | NA | NA | NA | NA | NA | NA | Anxiety not an outcome |
| Kerns, CM, Wood, JJ, Kendall, PC, Renno, P, Crawford, EA, Mercado, RJ, & Small, BJ 2016 | NA | N | NA | NA | NA | NA | NA | NA | NA | NA | NA | NA | NA | about the Rationale, Design and Methods for proposed research - no results |
| Kerns, CM, Collier, A, Lewin, AB, & Storch, EA 2017 | Y | Y | Y | N | N | Y | Y | U | Y | Y | Y | Y | Y | ( exclude - anxiety not main outcome although reduction reported) |
| Lundh, A, Forsman, M, Serlachius, E, Lichtenstein, P, & Landén, M 2013 | N | N | NA | NA | NA | NA | NA | NA | NA | NA | NA | NA | NA | secondary analysis of existing database; no autism |
| McConachie, H, & Mclaughlin, E 2011 | Y | U | U | N | N | U | U | U | Y | Y | Y | U | U | abstract only - unable to locate full paper |
| McNally Keehn, R 2010 | Y | Y | Y | N | N | Y | Y | Y | Y | Y | Y | Y | Y | same study as below |
| McVey, AJ, Dolan, BK, Willar, KS, Pleiss, S, Karst, JS, Casnar, CL, & Van Hecke, AV 2016 | NA | N | NA | NA | NA | NA | NA | NA | NA | NA | NA | NA | NA | 18-24 years - outside of 'school' age group of up to 18 years |
| Minshawi, NF, Wink, LK, Shaffer, R, Plawecki, MH, Posey, DJ, Liu, H, & Erickson, CA 2016 | Y | Y | Y | Y | Y | Y | Y | U | Y | Y | NA | Y | Y | Anxiety not an outcome (focus is on social skills - not strickly anxiety) |
| Reyes, NM 2009 | Y | N | NA | Y | NA | NA | NA | NA | NA | NA | NA | NA | NA | NA |
| Russell, AJ, Jassi, A, Fullana, MA, Mack, H, Johnston, K, Heyman, I, & Mataix‐Cols, D 2013 | NA | N | NA | Y | NA | NA | NA | NA | NA | NA | NA | NA | NA | Anxiety not primary outcome - OCD. mainly too old |
| Santomauro, D, Sheffield, J, & Sofronoff, K 2016 | Y | N | Y | N | N | U | Y | Y | Y | Y | Y | Y | Y | Poor quality |
| Scarpa, A, & Reyes, NM 2011 | NA | N | NA | Y | NA | NA | NA | NA | NA | NA | NA | NA | NA | NA |
| Schohl, KA, Van Hecke, AV, Carson, AM, Dolan, B, Karst, J, & Stevens, S 2014 | NA | N | NA | Y | NA | NA | NA | NA | NA | NA | NA | NA | NA | NA |
| Tachibana, Y, Hwang, Y, Abe, Y, Goto, S, Sugai, K, & Kawashima, R 2013 | Y | Y | Y | N | N | Y | Y | Y | Y | Y | NA | Y | Y | focused on enhancing executive function; managing anxiety is not the primary motive |
| Wood, JJ, Drahota, A, Sze, K, Van Dyke, M, Decker, K, Fujii, C, & Spiker, M 2009 | Y | Y | Y | N | N | Y | U | Y | Y | Y | Y | Y | Y | NA |
| Yoo, HJ, Bahn, G, Cho, IH, Kim, EK, Kim, JH, Min, JW, & Cho, S 2014 | Y | U | Y | N | N | N | U | Y | Y | Y | Y | Y | Y | NA |

## Appendix C: Critical appraisal checklist for quasi-experimental studies and results of excluded studies


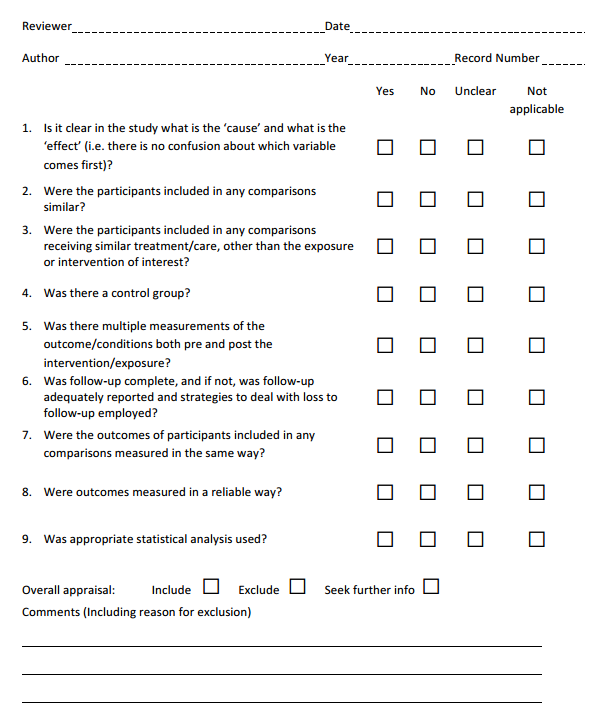


Source:
<http://joannabriggs.org/assets/docs/critical-appraisal-tools/JBI_Critical_Appraisal-Checklist_for_Quasi_-_Experimental_Studies.pdf>

| **Excluded Quasi-Experimental studies** | **Q1** | **Q2** | **Q3** | **Q4** | **Q5** | **Q6** | **Q7** | **Q8** | **Q9** | **Notes** |
| --- | --- | --- | --- | --- | --- | --- | --- | --- | --- | --- |
| Al Anbar NN, Dardennes RM, Prado-Netto A, Kaye K, & Contejean Y 2010 | NA | NA | NA | NA | NA | NA | NA | NA | NA | anxiety not an outcome, study is about parents of kids with ASD |
| Antshel, KM, Polacek, C, McMahon, M, Dygert, K, Spenceley, L, Dygert, L, & Faisal, F 2011 | Y | Y | Y | N | Y | U | Y | NA | NA | anxiety is comorbid, not a measured outcome |
| Azbell, E, & Laking, T 2006 | Y | Y | Y | Y | Y | U | Y | N | Y | observation of behaviours - not a standardised assessment |
| Baimbridge, EW 2013 | Y | N | U | Y | Y | Y | Y | Y | NA | descriptive stats only |
| Barry, TD, et al 2015 | Y | U | U | N | Y | U | Y | Y | NA | descriptive stats only |
| Berger, DS 2012 | Y | U | NA | N | NA | NA | NA | NA | NA | Anxiety not a measured outcome |
| Bitsika, V, & Sharpley, CF 2014 | Y | U | NA | N | N | NA | Y | Y | Y | correlation study - no treatment |
| Bitsika, V, & Sharpley, CF 2017 | NA | NA | NA | NA | NA | NA | NA | NA | NA | correlation study - no treatment |
| Bougher-Muckian, HR, Root, AE, Coogle, CG, & Floyd, KK 2016 | NA | U | NA | Y | NA | NA | NA | NA | NA | correlation study - no treatment |
| Burke, MK, Prendeville, P, & Veale, A 2017 | Y | U | NA | N | Y | NA | Y | Y | N | NA |
| Chiang, HL, & Gau, SSF 2016 | NA | NA | NA | Y | NA | NA | NA | NA | NA | correlation study - no treatment |
| Cipolla, DS 2013 | Y | NA | NA | NA | NA | NA | NA | NA | NA | only first 2 chaps of thesis and no mention of anxiety |
| Cotugno, AJ 2009 | Y | Y | Y | Y | Y | U | Y | Y | Y | NA |
| Curtin, C, Humphrey, K, Vronsky, K, Mattern, K, Nicastro, S, & Perrin, EC 2016 | Y | NA | NA | N | NA | NA | NA | NA | NA | pre-post design - no group |
| Danial, J 2013 | NA | NA | NA | NA | NA | NA | NA | NA | NA | single case |
| Deckers, A, Muris, P, Roelofs, J, & Arntz, A 2016 | NA | NA | NA | NA | NA | NA | NA | NA | NA | anxiety is a moderator, not an outcome |
| Drahota, AM 2008 | NA | NA | NA | NA | NA | NA | NA | NA | NA | full thesis - see published article |
| Drmic, IE, Aljunied, M, & Reaven, J 2017 | Y | NA | NA | N | Y | Y | NA | Y | Y | NA |
| Edgington, L, Hill, V, & Pellicano, E 2016 | Y | NA | NA | N | Y | Y | NA | Y | Y | NA |
| Ekman, E, & Hiltunen, AJ 2015 | Y | NA | NA | N | Y | Y | NA | Y | Y | NA |
| Epp, KM 2008 | NA | NA | NA | NA | NA | Y | NA | U | Y | Anxiety is not an outcome - just included under 'problem behavious' |
| Farrell, L, Waters, A, Milliner, E, & Ollendick, T 2012 | Y | Y | U | Y | Y | Y | Y | Y | Y | tentative - focus is on comorbid, but anxiety does appear to be an outcome measured |
| Fjermestad, KW, Vatne, TM, & Gjone, H 2015 | NA | NA | NA | N | NA | NA | NA | Y | Y | only 50% of participants have co-morbid ASD - I question that it meets our criteria |
| Gillis, JM, Hammond Natof, T, Lockshin, SB, & Romanczyk, RG 2009 | Y | NA | NA | N | Y | Y | NA | Y | U | Observational study? Note that term used is FEAR rather than anxiety - intervention not standardised across participants… |
| Goh, TJ, Sung, M, Ooi, YP, Lam, CM, Chua, A, Fung, D, & Pathy, P 2011 | NA | NA | NA | NA | NA | NA | NA | NA | NA | detail too brief, and pre-post |
| Hill, TL, Gray, SA, Baker, CN, Boggs, K, Carey, E, Johnson, C, & Varela, RE 2017 | Y | NA | NA | N | NA | NA | NA | NA | NA | NA |
| Hillier, AJ, Fish, T, Siegel, JH, & Beversdorf, DQ 2011 | NA | NA | NA | NA | NA | NA | NA | NA | NA | outside our age range (18 -24) |
| Hillier, A, Greher, G, Poto, N, & Dougherty, M 2012 | Y | NA | NA | N | NA | NA | NA | NA | NA | Most outside age criteria. No mention of being in school |
| Hillier, A, Greher, G, Queenan, A, Marshall, S, & Kopec, J 2016 | Y | NA | NA | Y | NA | NA | NA | NA | NA | Age 21 - 24 - outside our criteria |
| Hillier, A, Kopec, J, Poto, N, Tivarus, M, & Beversdorf, DQ 2016 | Y | NA | NA | N | NA | NA | NA | NA | NA | Aged between 13 and 29 years with an average of 18. |
| Hillier, A, Murphy, D, & Ferrara, C 2011 | Y | NA | NA | N | NA | NA | NA | NA | NA | Many outside our age range. |
| Johnco, CJ, De Nadai, AS, Lewin, AB, Ehrenreich-May, J, Wood, JJ, & Storch, EA 2015 | NA | NA | NA | NA | NA | NA | NA | NA | NA | This article is a synthesis of 4 other studies |
| Kaboski, JR, Diehl, JJ, Beriont, J, Crowell, CR, Villano, M, Wier, K, & Tang, K 2015 | Y | Y | U | Y | Y | Y | Y | Y | Y | NA - interesting study in that the comparison was between ASD and TD both going through treatment but TD group did not know their partner was ASD |
| Keefer, A, Kreiser, NL, Singh, V, Blakeley-Smith, A, Duncan, A, Johnson, C, & Vasa, RA 2017 | Y | NA | NA | N | NA | NA | NA | NA | NA | pre-post correlation study - no group |
| Klebanoff, S 2015 | N | U | Y | Y | U | U | Y | Y | U | bit hard to know what's going on in this study…random assignment of TD and ASD is confusing |
| Laugeson, EA, Ellingsen, R, Sanderson, J, Tucci, L, & Bates, S 2014 | Y | Y | Y | Y | Y | N | Y | Y | N | poor quality, eg anxiety parent report for 23% |
| Lei, J, Sukhodolsky, DG, Abdullahi, SM, Braconnier, ML, & Ventola, P 2017 | Y | N | N | N | Y | Y | NA | Y | Y | poor quality - observational at best |
| Lockwood, JA 2013 | NA | NA | NA | NA | NA | NA | NA | NA | NA | Proposal, study not actually conducted |
| Lordo, DN, Bertolin, M, Sudikoff, EL, Keith, C, Braddock, B, & Kaufman, DA 2017 | Y | Y | Y | Y | U | N | NA | Y | U | poor quality |
| Maddox, BB, Miyazaki, Y, & White, SW 2017 | N | NA | NA | NA | NA | Y | U | Y | Y | this is pre/post comparison - longitudinal design, and is based on the earlier RCT (see White, 2013) |
| Maskey, M, Lowry, J, Rodgers, J, McConachie, H, & Parr, JR 2014 | Y | NA | NA | N | NA | NA | NA | NA | NA | pre-post design |
| McGillivray, JA, & Evert, HT 2014 | Y | Y | U | Y | Y | Y | Y | Y | Y | No indication if older ones are still in school. Age outside criteria |
| Moskowitz, LJ, Walsh, CE, Mulder, E, McLaughlin, DM, Hajcak, G, Carr, EG, & Zarcone, JR 2017 | Y | NA | NA | N | NA | NA | NA | NA | NA | pre-post design |
| Murphy, D, Hillier, A, Ferrara, C, Baltisberger, N, & Lopes, A 2011 | Y | NA | NA | N | NA | NA | NA | NA | NA | age of 17.1 years (13-27 years) mainly too old |
| Ooi, YP, Lam, CM, Sung, M, Tan, WTS, Goh, TJ, Fung, DSS, & Chua, A 2008 | Y | NA | NA | N | NA | NA | NA | NA | NA | pre-post design |
| Ozsivadjian, A, & Knott, F 2011 | NA | NA | NA | N | NA | NA | NA | NA | NA | case-studies |
| Pourre, F, Andanson, J, Aubert, E, & Raynaud, JP 2013 | NA | NA | NA | N | NA | NA | NA | NA | NA | this is an abstract and looks Observational |
| Puleo, CM, & Kendall, PC 2011 | NA | NA | NA | NA | NA | NA | NA | NA | NA | this is TD with ASD symptomatology |
| Reaven, J, Blakeley-Smith, A, Leuthe, E, Moody, E, & Hepburn, S 2012 | NA | NA | NA | N | NA | NA | NA | NA | NA | Pre-post design - Files are in correct folders but titles need to be swapped. |
| Reaven J, Blakeley-Smith A, Beattie TL, Sullivan A, Moody, EJ, Stern, JA, & Smith, IM 2015 | Y | NA | NA | N | NA | NA | NA | NA | NA | main focus is on the clinician; pre-post design |
| Reaven, J, Washington, L, Moody, EJ, Stern, JA, Hepburn SL, & Blakeley-Smith A 2015 | NA | NA | NA | NA | NA | NA | NA | NA | NA | reduction of anxiety in parents not students |
| Rodgers, J, Hodgson, A, Shields, K, Wright, C, Honey, E, & Freeston, M 2017 | Y | NA | NA | U | N | NA | NA | NA | NA | intervention is for parents of kids with ASD; no clear comparison group; groups receiving different measures; pre-post design |
| Schohl, KA 2012 | Y | NA | NA | Y | NA | NA | NA | NA | NA | move to RCT for probable inclusion - this may be the published work of 2012 |
| Schohl, KA 2016 | Y | NA | NA | Y | NA | NA | NA | NA | NA | move to RCT for probable inclusion |
| Schoultz, P 2016 | Y | NA | NA | N | N | NA | NA | NA | NA | secondary analysis of Reaven ( 2015)and Storch et al., 2016 - but is looking at different aspects |
| Selles, RR 2013 | Y | NA | NA | N | NA | Y | NA | Y | NA | extent to which gains made during the course of a CBT treatment for anxiety in youth with ASD were extended beyond treatment end |
| Storch, EA, Nadeau, JM, Johnco, C, Timpano, K, McBride, N, Mutch, PJ, & Murphy, TK 2016 | Y | NA | NA | N | NA | NA | NA | NA | NA | correlation study |
| Storch, EA, Zavrou, S, Collier, AB, Ung, D, Arnold, EB, Mutch, PJ, & Murphy, TK 2015 | Y | U | U | U | NA | NA | NA | NA | NA | it suggests sub-group comparison but does not report any? Possibly reconsider for Observational |
| Van Dyke, M 2014 | Y | NA | NA | N | NA | NA | NA | NA | NA | mixed method study |
| van Steensel, FJ, Zegers, VM, & Bögels, SM 2017 | Y | NA | NA | N | NA | NA | NA | NA | NA | Good paper - but case-series |
| van Steensel, FJ, Bögels, SM, & Dirksen, CD 2012 | Y | U | U | Y | NA | NA | NA | Y | Y | NA |
| van Steensel, FJ, Dirksen, CD, & Bögels, SM 2014 | Y | Y | Y | Y | Y | NA | U | Y | Y | follow-on paper from main 2012 study - outcome is cost-effectiveness - there is anxiety reported but not the focus |
| Wentz, E, Nydén, A, & Krevers, B 2012 | Y | NA | NA | N | NA | NA | NA | NA | NA | ADHD and/or ASD, only 3 in our age range with ASD |
| White, SW, Schry, AR, Miyazaki, Y, Ollendick, TH, & Scahill, L 2015 | Y | NA | NA | N | NA | Y | NA | NA | NA | this is pre/post comparison - longitudinal design |
| Whitehead, JL 2005 | Y | NA | NA | N | NA | NA | NA | NA | NA | this is pre/post comparison - longitudinal design |
| Wright, H, Hall, S, Hames, A, Hardiman, J, Mills, R, PAWS Project Team, & Mills, D 2015 | Y | Y | U | Y | Y | Y | Y | Y | U | only collected pre-post anxiety in the intervention group so cant compare to control group |
| Yu, XT, Lam, HS, Au, CT, Chan, S, Chan, D, & Li, AM 2015 | Y | NA | NA | N | NA | NA | NA | NA | NA | longitudinal study |
| Zabel, JA 2015 | Y | NA | NA | N | NA | NA | NA | NA | NA | mixed method case study on 4 participants with inconclusive results |
| Zhang, R, Jia, MX, Zhang, JS, Xu, XJ, Shou, XJ, Zhang, XT, & Han, JS 2012 | Y | NA | Y | Y | Y | Y | Y | Y | Y | age 2-7 years mainly outside of our group |

## Appendix D: Data extraction instruments


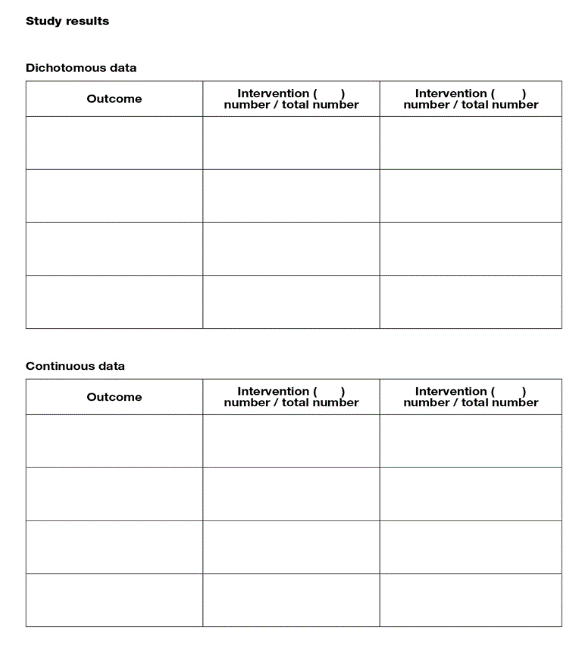


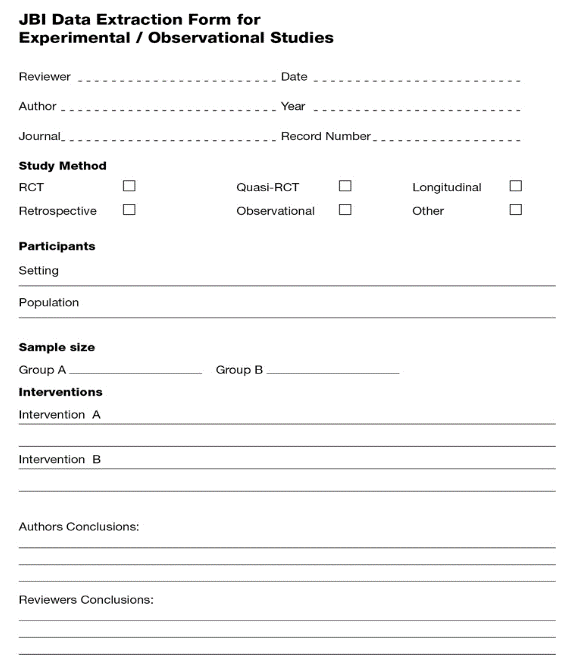

Supplement: Supplementary file 1 — Supporting information [file CL2-16-e1086-s001.docx]
